# Synthesis and Antileishmanial Evaluation of *N*-Phenyl-2-phenoxyacetamides Derived from Carvacrol

**DOI:** 10.1021/acsomega.4c11359

**Published:** 2025-04-23

**Authors:** Rayane Luiza de Carvalho, Alícia da Conceição Terbutino da Silva, Bianca Muniz Lacerda Ventura, Sara Andrade Machado Godoy, Olívia Géraldine Audrey Avome Nguema, Juliana Lopes Rangel Fietto, Christiane Mariotini-Moura, Michelle Dias de Oliveira Teixeira, Raphael de Souza Vasconcellos, Angel Amado Recio Despaigne, Bruna Vidal Paes, Bernardo Lages Rodrigues, Ulisses Alves Pereira, Patrícia Fontes Pinheiro

**Affiliations:** † Department of Biochemistry and Molecular Biology, Federal University of Viçosa, Avenida Peter Henry Rolfs, s/n, 36570-900 Viçosa, Minas Gerais, Brazil; ‡ Department of Chemistry, Federal University of Viçosa, Avenida Peter Henry Rolfs, s/n, 36570-900 Viçosa, Minas Gerais, Brazil; § Department of Medicine and Nursing, Federal University of Viçosa, Avenida Peter Henry Rolfs, s/n, 36570-900 Viçosa, Minas Gerais, Brazil; ∥ Department of Chemistry, Federal University of Minas Gerais, 1270-901 Belo Horizonte, Minas Gerais, Brazil; ⊥ Institute of Agricultural Sciences, Federal University of Minas Gerais, Montes Claros Regional Campus, Avenida Universitária 1000, Bairro Universitário, 39404-547 Montes Claros, Minas Gerais, Brazil

## Abstract

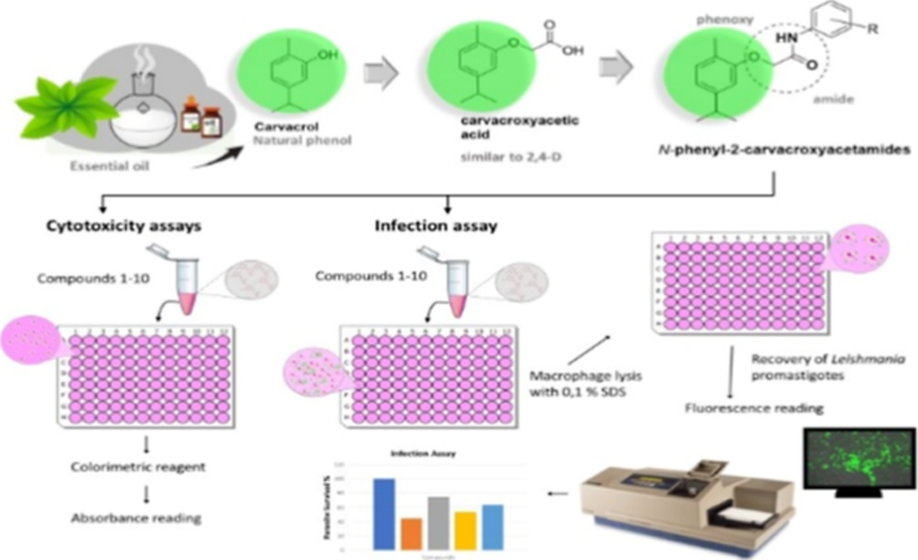

Leishmaniasis, caused by protozoa of the genus *Leishmania* and transmitted by sandflies, remains prevalent in underdeveloped countries. The disease manifests in three clinical forms: cutaneous, mucocutaneous, and visceral, the latter being fatal without adequate treatment. Current therapies, including pentavalent antimonials, have important limitations, such as high toxicity and reduced efficacy. This study investigates synthetic compounds derived from carvacrol as potential therapeutic alternatives. Carvacrol serves as a precursor for the synthesis of carvacroxyacetic acid, a key intermediate in producing *N*-phenyl-2-phenoxyacetamides. Ten novel molecules were synthesized with yields ranging from 11% to 60%. All synthesized compounds were characterized using mass spectrometry, ^1^H NMR and ^13^C NMR. Additionally, the structure of compound **9** (C_18_H_20_FNO_2_) was confirmed by X-ray crystallography. Cytotoxicity assays with macrophages and Vero cells revealed that all ten *N*-phenyl-2-phenoxyacetamides exhibited low or no toxicity at the tested concentrations. Compounds **5** and **7** demonstrated significant antileishmanial activity against *Leishmania braziliensis*, outperforming both the untreated control and the precursor carvacroxyacetic acid (CA). Furthermore, their efficacy against the parasite exceeded for the activity of pure carvacrol reported in the literature. These findings highlight the significant potential of these compounds and emphasize the importance of further studies to elucidate their mechanisms of action and explore broader therapeutic applications.

## Introduction

Leishmaniasis is a neglected anthropozoonotic disease that affects multiple countries across all continents,^1^ with approximately one billion people living in endemic regions. Among the various clinical manifestations, visceral leishmaniasis (VL) is the most lethal form, with around 30,000 new cases reported annually; and cutaneous leishmaniasis (CL) is the most common form, with over 1 million new cases reported each year.^2,3^

The disease is caused by more than 20 species of parasites of the genus *Leishmania*, which infect the host through the bite of infected female sandflies of the genera *Phlebotomus* and *Lutzomyia*. Clinical presentations vary according to the parasite species: *Leishmania infantum* and *Leishmania donovani* are primarily associated with the visceral form, whereas *Leishmania braziliensis* and *Leishmania amazonensis* predominate in cutaneous form.^4,5^

Treatment poses a challenge due to the limitations of the drugs currently in use. Medications such as pentavalent antimonials, amphotericin B, and miltefosine have significant drawbacks, including high toxicity, low efficacy, prolonged treatment duration, and the need for hospital administration, making patient compliance difficult.^6,7^

Amphotericin B, for instance, is one of the most recommended drugs for severe cases of VL; however, its dose-dependent nephrotoxicity limits its use.^8^ Adverse effects include renal insufficiency, hypokalemia, hypomagnesemia, metabolic acidosis, and polyuria caused by nephrogenic diabetes insipidus, necessitating caution when administering it to patients with renal disorders. Nephrotoxicity impacts treatment efficacy and reinforces the need for strategies to mitigate these adverse effects.^9,10^

Thus, there is significant interest in the discovery and development of new antileishmanial drugs, especially those with low toxicity to host cells. Compounds derived from natural products have garnered attention in research and industry due to their broad biological activity and lower toxicity.^11−14^

In this context, natural compounds serve as a valuable source of inspiration for the development of innovative therapeutic agents. Carvacrol, a phenolic monoterpene, is a significant natural derivative abundantly found in essential oils extracted from thyme, sage, rosemary, pepper, and oregano. Notably, carvacrol exhibits potent biological activities, including anticancer,^14^ antiviral,^15^ antileishmanial,^11^ antiinflammatory,^16^ antioxidant activities,^17^ among others.^18,19^

**Figure 1:**
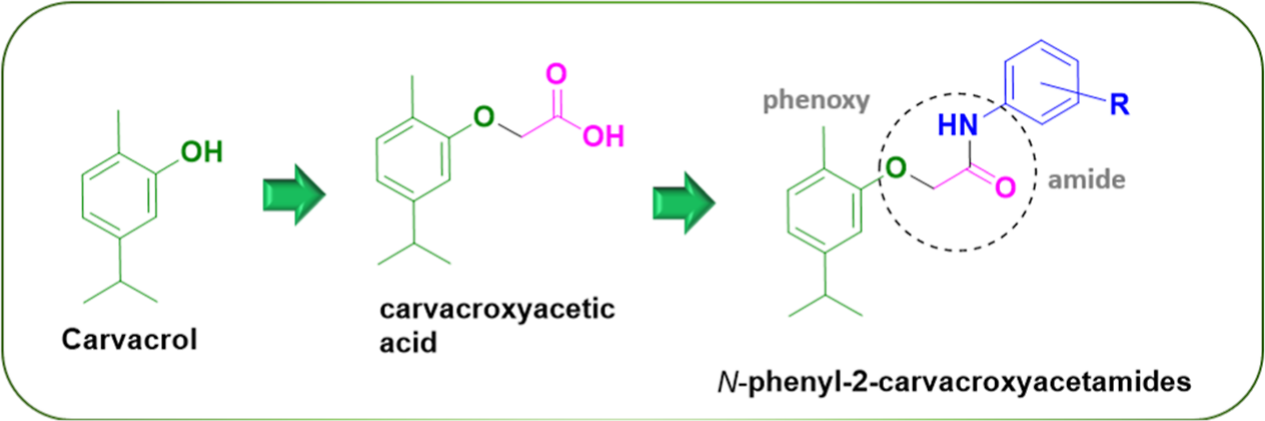
General structures of *N*-phenylcarvacroxyacetamides are derived from carvacroxyacetic acid synthesized from carvacrol (a natural phenol).

Carvacrol can be used to synthesize carvacroxyacetic acid, which serves as a key precursor for the production of amides, particularly *N*-phenyl-2-phenoxyacetamides (Figure 1). This class of compounds has demonstrated diverse bioactivities, including antimicrobial,^20,21^ antiviral (mainly against SARS-CoV-2),^22^ analgesic,^23^ antiinflammatory,^24^ insecticide,^25^ and herbicide properties.^26^ However, despite these promising biological activities, its potential antileishmanial activity remains unexplored to date.

Based on the antileishmanial properties and low toxicity associated with natural compounds, this study investigated the potential of semisynthetic derivatives of carvacrol for the development of new drugs that may provide alternative strategies to combat this important neglected disease.

Accordingly, ten novel molecules of *N*-phenyl-2-carvacroxyacetamides were synthesized and evaluated for cytotoxicity in mammalian cells and antileishmanial potential, aiming to identify an effective and low-toxicity compound for leishmaniasis treatment.

## Results and Discussion

### Synthesis

**Scheme 1:**
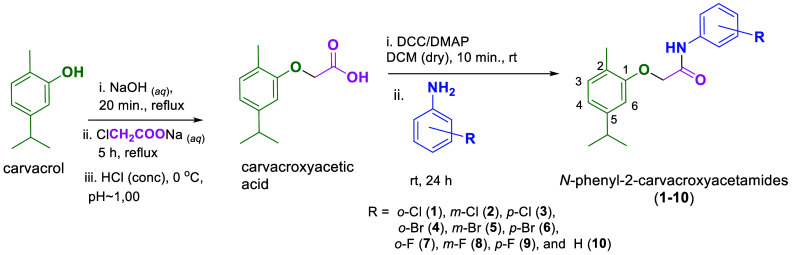
Synthetic Route to Obtain *N*-Phenyl-2-carvacroxyacetamides

*N*-Phenyl-2-carvacroxyacetamides were synthesized according to the synthetic route described in Scheme 1. In the initial step, carvacroxyacetic acid was prepared and subsequently used as the starting material for the synthesis of compounds **1–10** via Steglich amidation (DCC/DMAP) with anilines substituted at the *ortho*, *meta*, or *para* positions by Cl, Br, or F. Of these compounds, eight are new, while two (compounds **2** and **10**) had been previously reported by Patil et al.^20^

Carvacroxyacetic acid was obtained with a yield of 56%, closely matching the 59% yield reported by de Assis Alves et al.^19^ In the IR spectrum of carvacroxyacetic acid (Supporting Information), the O–H stretching band was observed in the range of 3300–2500 cm^–1^ consistent with the carboxylic acid functional group. Additionally, there was an intense band at 1735 cm^–1^ corresponding to the C=O bond of the same functional group.

Analysis of the mass spectrum of carvacroxyacetic acid revealed the molecular ion at *m*/*z* 208, corresponding to the compound’s molecular mass. The base peak was observed at *m*/*z* 149, indicating the loss of a methyl group (M^+•^ – 15), likely due to the fragmentation of the isopropyl group in the molecule.

In the ^1^H NMR spectrum of carvacroxyacetic acid, a signal at 4.53 ppm corresponding to the CH_2_ group (singlet, 2H) was observed. The spectrum also showed a signal at 174.5 ppm, attributed to carbonyl carbon (C=O). Other signals corresponding to the hydrogens of this compound were identified, confirming the successful preparation of this acid.

**Figure 2:**
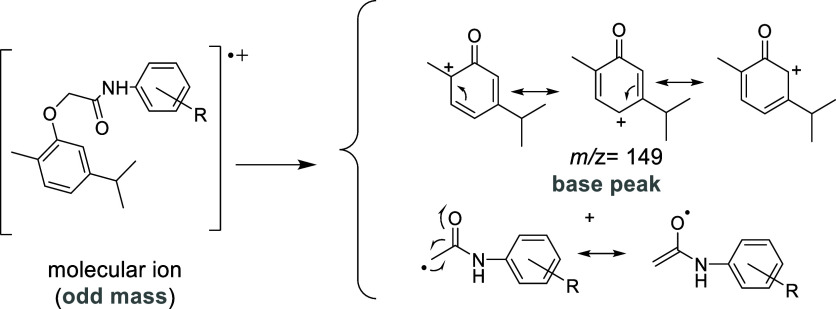
Proposed fragmentation pathway of *N*-phenyl-2-carvacroxyacetamides leading to the formation of the peak at *m*/*z* 149, which is the base peak in the mass spectra of compounds **1–10**.

Analysis of the mass spectra of compounds **1–10** revealed peaks with an odd mass for their molecular ions (Supporting Information). This odd mass is a consequence of the presence of a nitrogen atom in these compounds. Remarkably, the base peak in all spectra was at *m*/*z* 149, resulting from the fragmentation process favoring the formation of the radical cation associated with the carvacroxy group. The proposed fragmentation is presented in Figure 2.

In the ^1^H NMR spectra of *N*-phenyl-2-carvacroxyacetamides (**1–10**) (Supporting Information), a singlet was observed at δ_H_ 8.38–9.16 (1H), indicating the NH group. Additionally, another singlet was observed at δ_H_ 4.64–4.68 (2H), representing the CH_2_ group, a result similar to those reported by Pinheiro et al.^26^ for thymol-derived *N*-phenyl-2-phenoxyacetamides. Furthermore, signals corresponding to the protons in the two benzene rings and the protons linked to the sp^3^ carbons in the isopropyl and methyl groups, which are characteristic of carvacrol, were also identified.

The ^13^C NMR spectra for the compounds **1–10** (Supporting Information) showed signals for all the carbon atoms in these molecules. As an example, for compound **1**, the signal at δ_C_ 166.5 corresponded to the carbonyl carbon (C=O), the signal at δ_C_ 154.9 represented the carbon of the phenoxy group in the aromatic ring (CO), and the signals at δ_C_ 119.8–134.0 indicated the presence of carbons in the two benzene rings. An additional signal was observed at δ_C_ 67.3 for the CH_2_ group, and for the sp^3^ carbons, signals were observed at δ_C_ 34.1 (CH), 24.0 (CH(CH_3_)_2_), and 16.2 (CH_3_).

Based on these data, all *N*-phenyl-2-carvacroxyacetamides (**1–10**) were characterized, and subsequently utilized in the preparation of suspensions using DMSO solution in water for use in the biological assay.

### X-ray Diffraction Analysis

**Figure 3:**
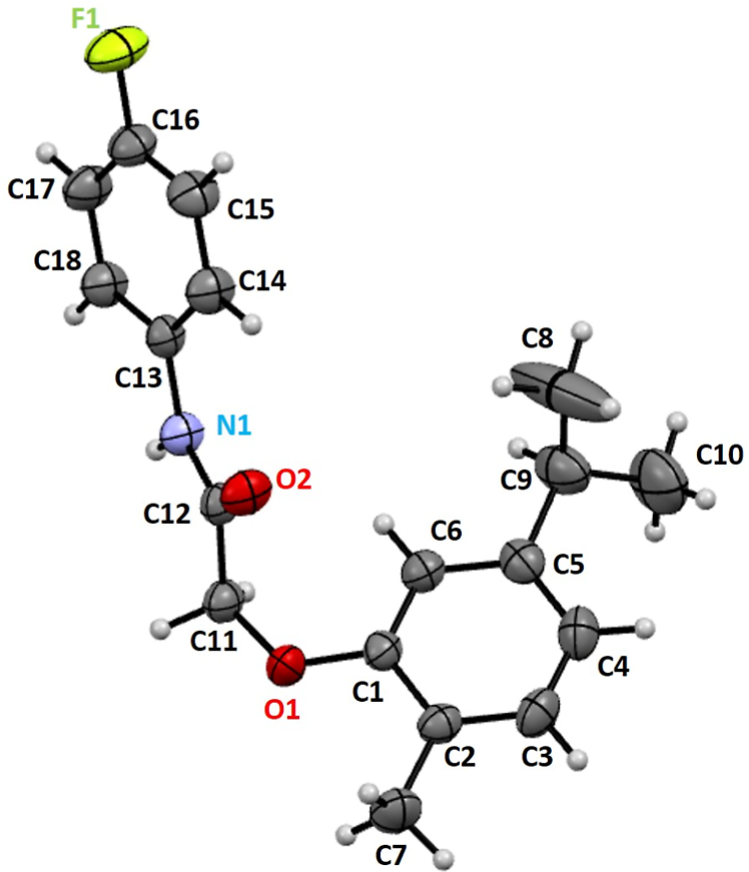
Ortep representation of C_18_H_20_FNO_2_ showing the labeling scheme of the nonH atoms with vibration ellipsoids at 50% level.

The perspective view of C_18_H_20_FNO_2_ is shown in Figure 3. Crystal data and refinement results for the determined structure are reported in Table S1 (Supporting Information).

Selected intramolecular bond distances and angles for the structure are given in Table 1. Carbon–oxygen bond distances are as expected: C12–O2 bond distance of 1.226(2) Å indicates the presence of the double bond. C11–O1 bond distance of 1.412(2) Å reflects the single bond character for this interaction, while the somehow shorter distance of 1.379(2) Å for C1–O1 bonding reflects the sp^2^ hybridization of carbon C1. The bond distance of 1.369(2) Å for C1–F1 is as expected. The bond angles for C1–O1–C11, N1–C12–C11, and C12–N1–C13 were observed in 118.2(14), 113.19(14), and 127.83(14)°, respectively.

**Table 1:** Selected Bond Lengths [Å] and Angles [deg] for the Structure

atoms	lengths [Å]	atoms	angles [deg]
N1–C12	1.350(2)	C12–N1–C13	127.83(14)
N1–C13	1.414(2)	C1–O1–C11	118.24(14)
O1–C1	1.379(2)	O2–C12–N1	124.58(16)
O1–C11	1.412(2)	O2–C12–C11	122.23(16)
F1–C16	1.369(2)	N1–C12–C11	113.19(14)
C12–O2	1.226(2)	C8–C9–C10	111.8(3)

**Figure 4:**
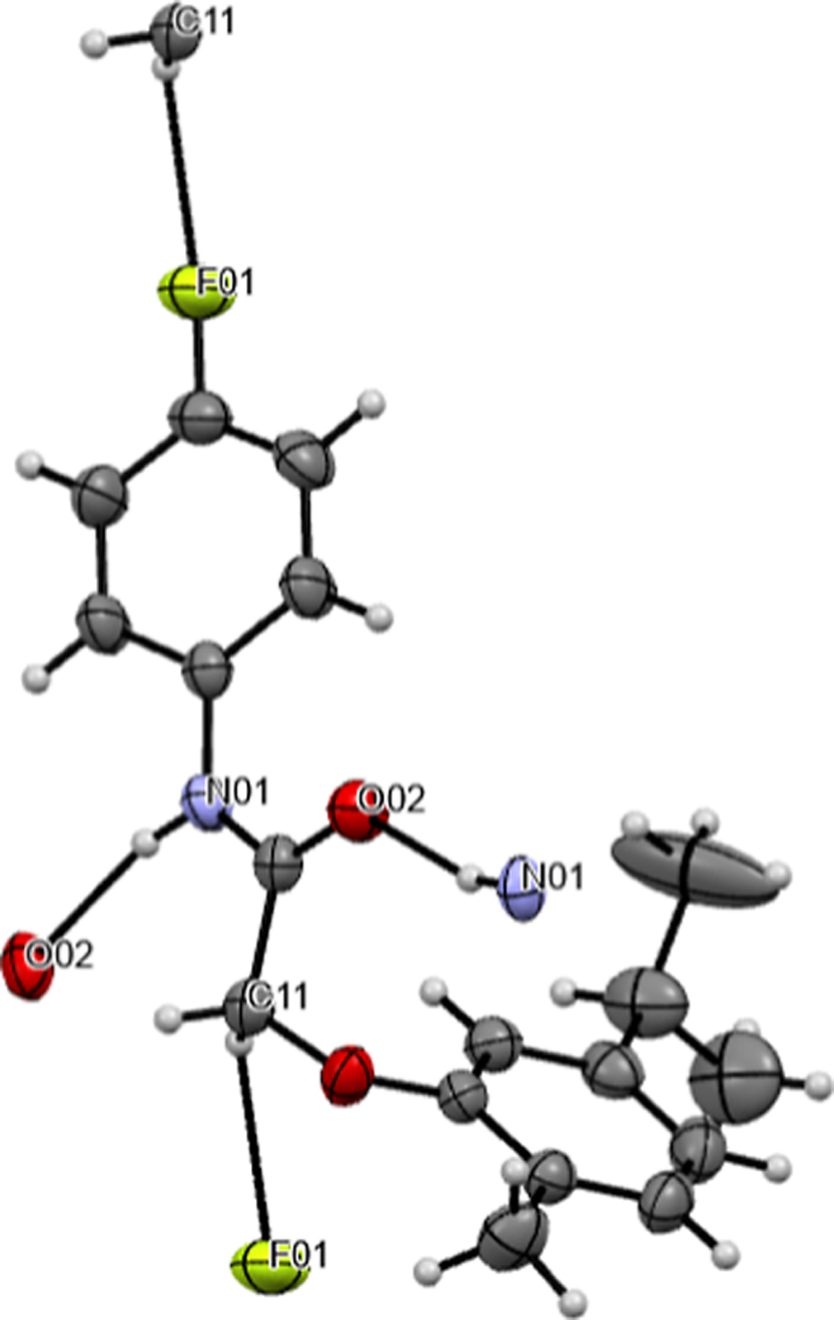
Molecular packing of C_18_H_20_FNO_2_ showing the intermolecular hydrogen bonds.

A weak intramolecular C14–H···O2 hydrogen bond (see Table 2) is present in the structure (Figure 2). In the packing of the molecule, two intermolecular N1–H···O2 and C11–H···F1 hydrogen bonds occur (see Figure 4 and Table 2).

**Table 2:** Hydrogen Bond Distances [Å] and Angles [deg] for C_18_H_20_FNO_2_ with *d*(H···A) < *r*(Å) + 2.00 Å and ⟨D–H···A⟩ 110°

D–H···A	*d*(D–H)	*d*(H···A)	*d*(D···A)	(D–H···A)	symmetry operation
C14–H···O2	0.93	2.33	2.89	118.7	intramolecular
N1–H···O2	0.83	2.05	2.87	166.7	[−*x* + *y*, −x + 1, *z* – 1/3]
C11–H···F1	0.97	2.60	3.37	136.0	[*x* + 1, *y* + 1, *z*]

### Cytotoxicity Assay in Macrophage RAW 264.7

Macrophages are key cells in the infection of vertebrate hosts by *Leishmania*. After being transmitted through the bite of a phlebotomine sandfly, promastigotes are phagocytosed by neutrophils, dendritic cells, and macrophages, where they differentiate into amastigotes and replicate within the intracellular environment. Due to the ease of cultivating macrophages in the laboratory, these cells represent a relevant biological model for studying the parasite-vertebrate host interaction, and numerous studies have employed them in infection models.^27−29^

Candidate drugs for the treatment of leishmaniasis must be capable of eliminating parasites within macrophages without compromising the viability of these cells. Thus, cytotoxicity assays in macrophages are crucial to determine whether a compound exhibits selective toxicity, meaning its ability to destroy the parasites while preserving the integrity of the host cell.^27^

**Figure 5:**
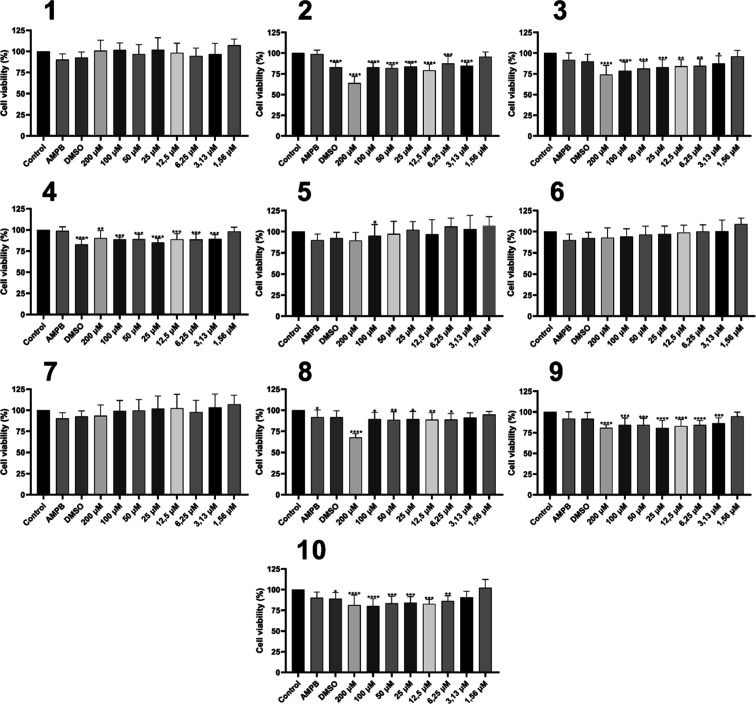
Cytotoxicity profile in RAW 264.7 macrophages for compounds **1–10**. Data are presented as the mean of three independent experiments. *p < 0.05, **p < 0.01, ***p < 0.001, ****p < 0.0001.

Cytotoxicity assays in RAW 264.7 cells demonstrated that compounds **1**–**10** exhibit low levels of toxicity at high concentrations, with CC_50_ values exceeding 200 μM (Figure 5), comparable to those reported for carvacrol itself in the literature. According to studies conducted by Costa et al.,^11^ the use of carvacrol in cytotoxicity tests on murine peritoneal macrophages yielded CC_50_ values of 75.91 μg mL^–1^ (equivalent to 505.3 μM) with 24 h treatment and CC_50_ = 40.23 μg mL^–1^ (equivalent to 267.8 μM) with 72 h treatment. Galvão et al.^12^ tested the cytotoxicity of carvacrol on a human monocyte cell line, reporting a CC_50_ value of 64.10 μg mL^–1^ (equivalent to 426.8 μM) with 48 h treatment. Additionally, due to solubility issues with the tested compound, Galvão et al.^12^ investigated whether the cytotoxicity of carvacrol could be reduced using a lipid nanocapsule encapsulation method. However, the value obtained was only CC_50_ = 73.50 μg mL^–1^ (equivalent to 489.5 μM) with 48 h treatment, indicating no significant changes. Therefore, it can be concluded that the semisynthetic compounds exhibited cytotoxicity similar to those previously tested. For some compounds, such as **1**, **5**, **6**, and **7**, no significant differences were observed at the highest concentration compared to the control, suggesting that testing at higher concentrations could yield CC_50_ values even higher than those reported in the literature.

### Cytotoxicity Assay in Vero Cells

Vero cells, derived from renal epithelial cells of the African green monkey (*Cercopithecus aethiops*), are widely used in cytotoxicity assays.^30−32^

Although Vero cells are not commonly used in *Leishmania* sp. infection models, studies have shown that *Leishmania chagasi* can invade these cells, which may represent a less hostile environment compared to macrophages.^33^ Pessotti and collaborators demonstrated that *L. chagasi* promastigotes can adhere to Vero cells and differentiate into amastigotes.^33^

Even liposomal formulation of amphotericin B that is generally well-tolerated present cases of hypokalemia and nephrotoxicity as the most frequently adverse effects reported.^34^ Among acute tubular injuries, toxic lesions are noteworthy, often caused by medications. Proximal tubular epithelial cells and those in the ascending limb of loop of Henle, being more absorptive, are more susceptible to damage, representing the second leading cause of acute kidney injury. Studies using Vero cells and mouse models have been reported in the literature for the evaluation of nephrotoxicity.^35,36^

**Figure 6:**
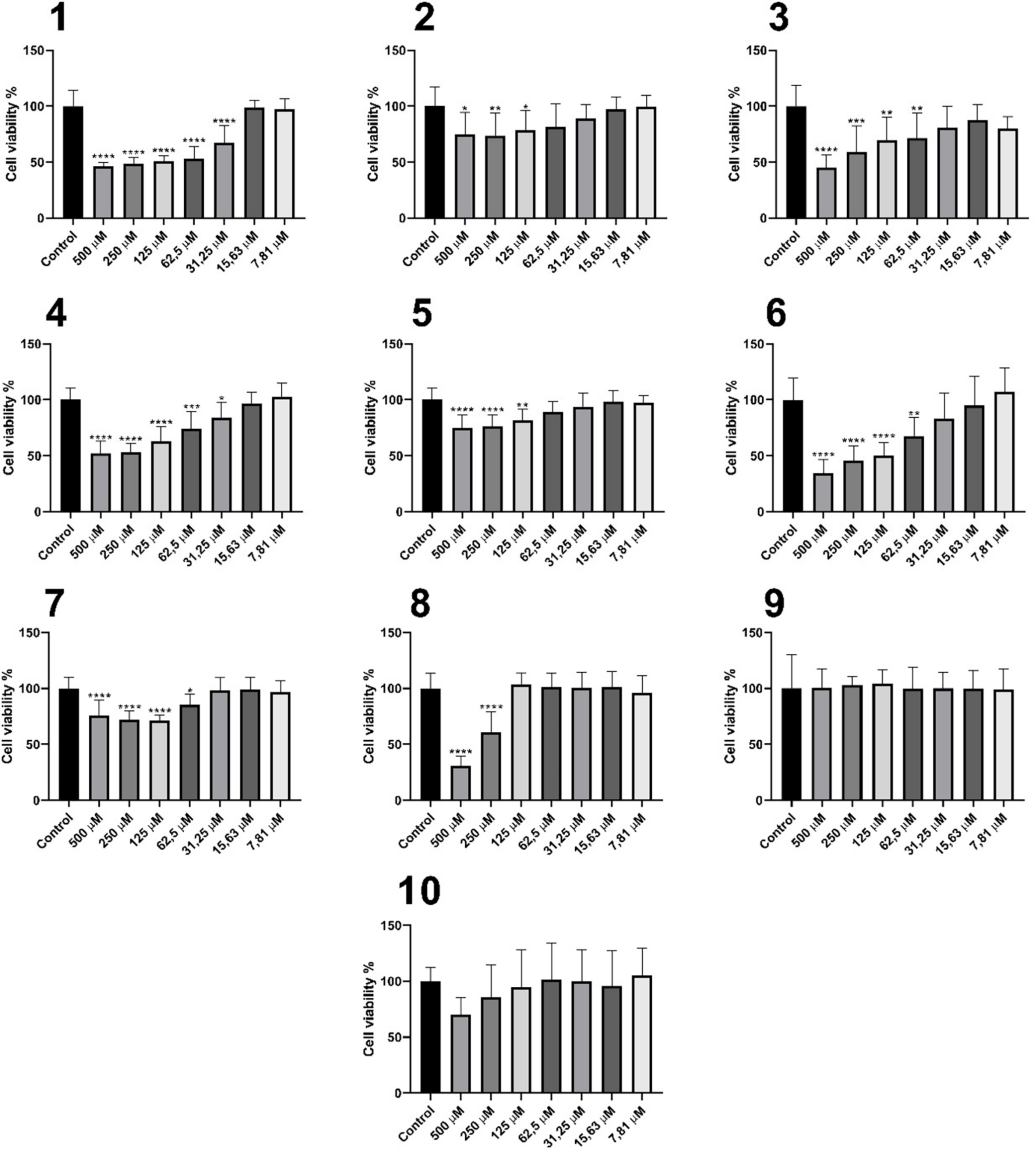
Cytotoxicity profile in Vero cells for compounds **1–10**. Data are presented as the mean of three independent experiments. *p < 0.05, **p < 0.01, ***p < 0.001, ****p < 0.0001.

Cytotoxicity assays in Vero cells (Figure 6) demonstrated pronounced toxicity at high concentrations (500 and 250 μM), with survival rates below 50% for treatments with molecules **1**, **3**, **4**, **6**, and **8**, exhibiting CC_50_ values of 200.3, 449.4, 372.1, 181.8, and 337 μM, respectively. As the concentration decreases, mild or undetectable toxicity is observed, evidenced by increased cell viability comparable to the control. Treatments with molecules **2**, **5**, **7**, **9**, and **10** maintained survival rates above 50% at all tested concentrations.

Nakamura de Vasconcelos and colleagues^37^ estimated the CC_50_ of carvacrol in Vero cells to be 86 ± 1.41 μg mL^–1^ (equivalent to 572.5 μM) when evaluated in 5 × 10^4^ cells per well, indicating lower toxicity compared to derivatives **1**, **3**, **4**, **6**, and **8**. However, the number of cells used in their analyses was five times higher than that used in the present study.

Treatments with molecules **1–10** at concentrations equal to or below 15.63 μM showed no direct harmful effects on cells under these conditions. Additionally, França and colleagues demonstrated that amphotericin B exhibits over 70% toxicity in Vero cells at 15 μg mL^–1^ (equivalent to 16.23 μM) through a neutral red cytotoxicity assay.^100^ Thus, based on these data, compounds **1–10** showed less detrimental effects on Vero cells compared to one of the main antileishmanial drugs.

### In Vitro Antileishmanial Activity

**Figure 7:**
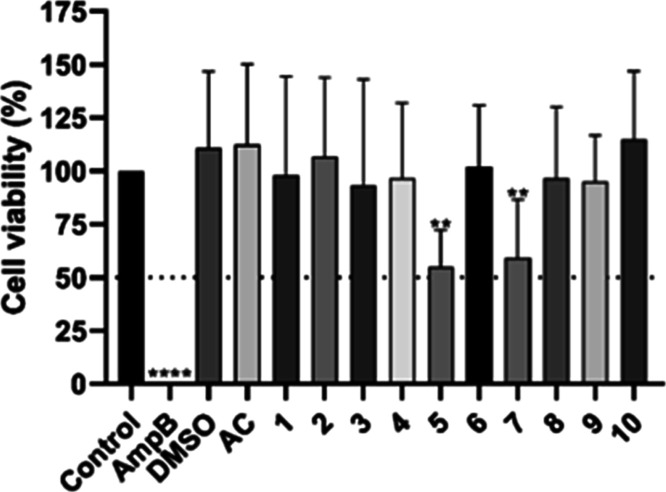
Screening of in vitro infection of RAW 264.7 macrophages by *L. braziliensis* MHOM/BR/75/M2904—GFP. AmpB—positive control of amphotericin B (3.125 μg mL^–1^), DMSO—negative control of dimethyl sulfoxide (0.04%). The data are presented as means of three independent experiments. **p < 0.01, ****p < 0.0001.

After confirming the low toxicity to macrophages and Vero cells, we evaluated compounds **1**–**10** for antileishmanial activity, as illustrated in Figure 7. The aim was to evaluate whether the derived compounds would exhibit higher activity compared to their direct precursor, carvacroxyacetic acid (AC). As observed, compounds **5** and **7** showed significant differences compared to the control, with parasite viability remaining around 50% at the tested screening concentration of 10 μM.

The results presented were highly promising. According to the literature, the IC_50_ values obtained in assays with *L. amazonensis* were approximately 27.40 μg mL^–1^ (182.4 μM) and 19.65 μg mL^–1^ (130.8 μM) for pure and encapsulated carvacrol, respectively, following 48 h treatments of parasites in the amastigote stage.^12^ In another study that also employed *L. amazonensis*, the IC_50_ values ranged from 37 μg mL^–1^ (246.4 μM) to 58 μg mL^–1^ (386.2 μM) for hydrogels containing carvacrol administered over 24 h treatments on murine peritoneal macrophages infected with *Leishmania*, and from 29 μg mL^–1^ (193.1 μM) to 42 μg mL^–1^ (279.7 μM) for 72 h treatments.^11^ Thus, the compounds under investigation appear to be highly promising, as a treatment at 10 μM resulted in the death of approximately 50% of the parasites. This observation suggests that the IC_50_ values for compounds **5** and **7** could be around 10 μM, which is 10-fold lower than those reported in the literature, indicating enhanced selectivity for *L. braziliensis*. Combined with the low cytotoxicity, this finding allows us to increase the concentration of the compounds in infection assays without increasing toxicity to host cells.

Thus, we present cytotoxicity assays using two distinct mammalian cell lines (macrophages and Vero cells), which demonstrated that all 10 novel semisynthetic compounds exhibited low or no toxicity at the tested concentrations. This finding is consistent with the literature and underscores the potential of these compounds for future studies. Regarding the antileishmanial potential of compounds **5** and **7**, the results were significant when compared to the untreated cell control and the precursor carvacroxyacetic acid (AC), demonstrating that the derivatives exhibit superior antileishmanial activity. Furthermore, they also surpassed that reported in the literature for pure carvacrol.

In summary, our findings emphasize the viability of exploring such derivatives as alternative treatments for leishmaniasis, a disease that remains a significant public health challenge. Future studies should focus on elucidating the mechanisms of action of these compounds, optimizing their chemical structures to enhance efficacy, and evaluating their performance in in vivo models. Additionally, assessing their pharmacokinetic and pharmacodynamic profiles will be essential for advancing these candidates toward clinical applications. By addressing these aspects, this research paves the way for the development of safer and more effective therapies for leishmaniasis.

## Methods

### General Experimental Procedures

All solvents were reagent grade and were used as received. Carvacrol, 2-chloroacetic acid, NaOH, HCl, aniline, substituted anilines (*o*, *m*, *p*-Br, -Cl, and -F), diethyl ether, dichloromethane, acetone, hexane, and ethyl acetate were purchased from Sigma-Aldrich.

For thin-layer chromatography (TLC) analysis, silica gel-coated aluminum sheets (250 μm thick) were utilized. Subsequently, the TLC plates were observed under ultraviolet light (254 nm) and then stained with either an ethanolic solution of phosphomolybdic acid or an aqueous solution of potassium permanganate.

Column chromatography separations were conducted using silica gel (70–230 mesh) as the stationary phase, with a mixture of hexane and ethyl acetate in a 5:1, v/v ratio used as the eluent.

Melting point temperatures were determined using an MQAPF-302 device and were uncorrected. The infrared spectra were obtained using a Varian 660-IR instrument employing the attenuated total reflectance (ATR) technique, scanning from 400 to 4000 cm^–1^.

The mass spectra were obtained on a Shimadzu GCMS-QP2010C Ultra mass spectrometer at the Center for Chemical Analysis of Environmental and Agroindustrial Samples (NAQAA) at UFV.

The ^1^H and ^13^C nuclear magnetic resonance analyses were carried out at LAREMAR—Nuclear Magnetic Resonance Laboratory, Department of Chemistry, UFMG. Deuterated chloroform (CDCl_3_) from Sigma-Aldrich was used as the solvent, and tetramethylsilane (TMS) was used as the internal reference standard (δ_H_ = 0). Scalar coupling constants (*J*) were expressed in hertz.

### Synthesis of Carvacroxyacetic Acid

The methodology used to synthesize the carvacroxyacetic acid from carvacrol was conducted in accordance with the literature.^19^ The characterization data for carvacroxyacetic acid, including infrared spectrum, mass spectrum, and ^1^H and ^13^C NMR spectra, are consistent with previously published data.

### General Method for the Synthesis of Compounds **1–10**

#### Exemplified by the Synthesis of *N*-(2-Chlorophenyl)-2-(5-isopropyl-2-methylphenoxy)acetamide (**1**)

In a double-necked round-bottom flask (25 mL) containing anhydrous calcium chloride and sealed with cotton wool, carvacroxyacetic acid (0.96 mmol, 0.200 g), *N*,*N*′-dicyclohexylcarbodiimide (DCC) (1.04 mmol, 0.210 g), 4-dimethylaminopyridine (DMAP) (0.096 mmol, 0.0110 g), and 5 mL of anhydrous dichloromethane (DCM) were combined. This mixture was magnetically stirred at room temperature for 5 min (Scheme 1). In a separate beaker, *o*-chloroaniline (0.80 mmol, 84 μL) was dissolved in 5 mL of dichloromethane and gradually added, drop by drop, to the round-bottom flask containing the reagent mixture. After this step, the reaction was maintained under magnetic stirring at room temperature for 24 h (following the procedure of Pinheiro et al.).^26^ The resulting compound was purified by column chromatography, using silica gel as the stationary phase and a mixture of hexane/ethyl acetate (5:1 v/v) as the mobile phase. The synthesis of the other *N*-phenyl-2-carvacroxyacetamides (**2–10**) followed the same methodology.

#### *N*-(2-Chlorophenyl)-2-(5-isopropyl-2-methylphenoxy)acetamide (**1**)

White crystalline solid (56 mg, 22% yield), mp: 80.4–82.1 °C. NMR, δ_H_ (400 MHz, CDCl_3_): 1.27 (d, 6H, *J* = 8.0 Hz, 2× CH_3_); 2.39 (s, 3H, CH_3_); 2.92 (sept, 1H, *J* = 8.0 Hz, CH); 4.69 (s, 2H, CH_2_); 6.75 (s, 1H, Ar-H_6_); 6.88 (d, 1H, *J* = 8.0 Hz, Ar-H_3_); 7.09–7.17 (m, 2H, H_3_ and H_4′_); 7.34 (t, 1H, *J* = 8.0 Hz, H_5′_); 7.43 (d, 1H, *J* = 8.0 Hz, H_6′_); 8.54 (d, 1H, *J* = 8.0 Hz, H_3′_); 9.16 (s, 1H, NH). δ_C_ (100 MHz, CDCl_3_): 16.23 (CH_3_); 24.1 (CH(CH_3_)_2_); 34.1 (CH(CH_3_)_2_); 67.3 (O–CH_2_); 109.7 (C_6_); 119.9 (C_4_); 121.3 (C_6′_); 123.0 (C_4′_); 123.8 (C_2_); 125.0 (C_2′_); 127.9 (C_5′_); 129.2 (C_3′_); 131.0 (C_3_); 134.0 (C_1′_); 148.5 (C_5_); 154.9 (C_1_); 166.5 (C=O). MS, *m*/*z* (%): 319 ([M + 2]^+•^, 12); 317 (C_18_H_20_ClNO_2_, [M]^+•^, 37); 281 (11); 207 (26); 190 (16); 163 (24); 149 (100); 127 (40); 105 (29); 91 (38); 77 (27); 41 (7).

#### *N*-(3-Chlorophenyl)-2-(5-isopropyl-2-methylphenoxy)acetamide (**2**)

White crystalline solid (28 mg, 11% yield), mp: 74.9–76.7 °C. NMR, δ_H_ (400 MHz, CDCl_3_): 1.27 (d, 6H, *J* = 8.0 Hz, 2× CH_3_); 2.35 (s, 3H, CH_3_); 2.91 (sept, 1H, *J* = 8.0 Hz, CH); 4.65 (s, 2H, CH_2_); 6.74 (s, 1H, Ar-H_6_); 6.89 (d, 1H, *J* = 8.0 Hz, Ar-H_4_); 7.16 (d, 1H, *J* = 8.0 Hz, H_3_); 7.35–7.26 (m, 2H, H_5′_ and H_6′_); 7.46 (dt, *J* = 8.4, 1.3 Hz, 1H, H_4′_); 7.74 (t, *J* = 2.1 Hz, 1H, H_2′_); 8.41 (s, 1H, NH). δ_C_ (100 MHz, CDCl_3_): 16.0 (CH_3_); 24.0 (CH(CH_3_)_2_); 34.0 (CH(CH_3_)_2_); 67.8 (O–CH_2_); 110.5 (C_6_); 117.9 (C_4_); 120.0 (C_6′_); 120.2 (C_2′_); 123.7 (C_2_); 124.9 (C_4′_); 131.1 (C_3_); 131.2 (C_5′_); 134.8 (C_3′_); 138.0 (C_1′_); 148.8 (C_5_); 155.1 (C_1_); 166.6 (C=O). MS, *m*/*z* (%): 319 ([M + 2]^+•^, 12); 317 (C_18_H_20_ClNO_2_, [M]^+•^, 35); 190 (19); 163 (24); 149 (100); 133 (22); 105 (27); 91 (32); 77 (15); 41 (8).

#### *N*-(4-Chlorophenyl)-2-(5-isopropyl-2-methylphenoxy)acetamide (**3**)

White crystalline solid (109 mg, 43% yield), mp: 87.5–89.2 °C. NMR, δ_H_ (400 MHz, CDCl_3_): 1.27 (d, 6H, *J* = 8.0 Hz, 2× CH_3_); 2.35 (s, 3H, CH_3_); 2.90 (sept, 1H, *J* = 8.0 Hz, CH); 4.65 (s, 2H, CH_2_); 6.74 (s, 1H, Ar-H_6_); 6.89 (d, 1H, *J* = 8.0 Hz, Ar-H_4_); 7.16 (d, 1H, *J* = 8.0 Hz, H_3_); 7.35 (d, 2H, *J* = 8.0 Hz, H_3′_ and H_5′_); 7.56 (d, 2H, *J* = 8.0 Hz, H_2′_ and H_6′_); 8.41 (s, 1H, NH). δ_C_ (100 MHz, CDCl_3_): 16.0 (CH_3_); 24.0 (CH(CH_3_)_2_); 34.0 (CH(CH_3_)_2_); 67.8 (O–CH_2_); 110.5 (C_6_); 120.2 (C_4_); 121.9 (C_2′_ and C_6′_); 123.4 (C_2_); 128.9 (C_3′_ and C_5′_); 131.1 (C_3_); 135.5 (C_1′_ and C_4′_); 148.8 (C_5_); 155.1 (C_1_); 166.6 (C=O). MS, *m*/*z* (%): 319 ([M + 2]^+•^, 3); 317 (C_18_H_20_ClNO_2_, [M]^+•^, 9); 190 (20); 163 (26); 149 (100); 127 (37); 105 (29); 91 (33); 77 (14); 65 (5); 41 (7).

#### *N*-(2-Bromophenyl)-2-(5-isopropyl-2-methylphenoxy)acetamide (**4**)

White crystalline solid (64 mg, 22% yield). mp: 94.1–96.0 °C. NMR, δ_H_ (400 MHz, CDCl_3_): 1.28 (d, 6H, *J* = 6.9 Hz, 2× CH_3_); 2.41 (s, 3H, CH_3_); 2.92 (sept, 1H, *J* = 6.9 Hz, CH); 4.70 (s, 2H, CH_2_); 6.76 (s, 1H, Ar-H_4_); 6.88 (dd, *J* = 7.6, 1.6 Hz, 1H, Ar-H_6_); 7.05 (td, *J* = 7.7, 1.6 Hz, 1H, Ar-H_3_); 7.16 (d, *J* = 7.6 Hz, 1H, H_4′_); 7.38 (td, *J* = 7.8, 1.5 Hz, 1H, H_5′_); 7.60 (dd, *J* = 8.1, 1.4 Hz, 1H, H_6′_); 8.52 (dd, *J* = 8.3, 1.6 Hz, 1H, H_3′_); 9.10 (s, 1H, NH). δ_C_ (100 MHz, CDCl_3_): 16.5 (CH_3_); 24.1 (CH(CH_3_)_2_); 34.1 (CH(CH_3_)_2_); 67.4 (O–CH_2_); 109.8 (C_6_); 113.4 (C_2′_); 119.9 (C_4_); 121.7 (C_3_); 123.9 (C_2_); 128.5 (C_6′_); 130.0 (C_5′_); 131.1 (C_4′_); 132.4 (C_3′_); 135.1 (C_1′_); 148.5 (C_5_); 155.0 (C_1_); 166.6 (C=O). MS, *m*/*z* (%): 363 (C_18_H_20_BrNO_2_, [M + 2]^+•^, 23); 361 ([M]^+•^, 24); 282 (9); 240 (2); 163 (28); 149 (100); 121 (22); 105 (41); 91 (45); 77 (19); 41 (9).

#### *N*-(3-Bromophenyl)-2-(5-isopropyl-2-methylphenoxy)acetamide (**5**)

White crystalline solid (44 mg, 15% yield), mp: 94.1–95.7 °C. NMR, δ_H_ (400 MHz, CDCl_3_): 1.26 (d, 6H, *J* = 8.0 Hz, 2× CH_3_); 2.35 (s, 3H, CH_3_); 2.90 (sept, 1H, *J* = 8.0 Hz, CH); 4.65 (s, 2H, CH_2_); 6.73 (s, 1H, Ar-H_6_); 6.91 (d, 1H, *J* = 8.0 Hz, Ar-H_4_); 7.16 (d, 1H, *J* = 8.0 Hz, H_3_); 7.23–7.33 (m, 2H, H_4′_ and H_5′_); 7.53 (d, 1H, *J* = 8.0 Hz, H_6′_); 7.87 (s, 1H, H_2′_); 8.39 (s, 1H, NH). δ_C_ (100 MHz, CDCl_3_): 16.0 (CH_3_); 24.0 (CH(CH_3_)_2_); 34.0 (CH(CH_3_)_2_); 67.8 (O–CH_2_); 110.4 (C_6_); 118.4 (C_4_); 120.2 (C_6′_); 122.8 (C_3′_); 123.8 (C_2′_); 127.9 (C_2_); 130.4 (C_4′_); 131.1 (C_3_ and C_5′_); 138.2 (C_1′_); 148.8 (C_5_); 155.1 (C_1_); 166.7 (C=O). MS, *m*/*z* (%): 363 (C_18_H_20_BrNO_2_, [M + 2]^+•^, 24); 361 ([M]^+•^, 24); 190 (17); 163 (25); 149 (100); 91 (37); 77 (12); 41 (8).

#### *N*-(4-Bromophenyl)-2-(5-isopropyl-2-methylphenoxy)acetamide (**6**)

White crystalline solid (89 mg, 31% yield), mp: 101.7–102.3 °C. NMR, δ_H_ (400 MHz, CDCl_3_): 1.26 (d, 6H, *J* = 8.0 Hz, 2× CH_3_); 2.35 (s, 3H, CH_3_); 2.90 (sept, 1H, *J* = 8.0 Hz, CH); 4.64 (s, 2H, CH_2_); 6.73 (s, 1H, Ar-H_6_); 6.89 (d, 1H, *J* = 8.0 Hz, Ar-H_4_); 7.16 (d, 1H, *J* = 4.0 Hz, Ar-H_3_); 7.48–7.54 (m, 4H, H_2′_, H_3′_, H_5′_, and H_6′_); 8.40 (s, 1H, NH). δ_C_ (100 MHz, CDCl_3_): 16.0 (CH_3_); 24.1 (CH(CH_3_)_2_); 34.0 (CH(CH_3_)_2_); 67.8 (O–CH_2_); 110.5 (C_6_); 117.5 (C_4′_); 120.2 (C_4_); 121.5 (C_2′_ and C_6′_); 123.7 (C_2_); 131.1 (C_3_); 132.1 (C_3′_ and C_5′_); 136.0 (C_1′_); 148.8 (C_5_); 155.1 (C_1_); 166.6 (C=O). MS, *m*/*z* (%): 363 (C_18_H_20_BrNO_2_, [M + 2]^+•^, 30); 361 ([M]^+•^, 30); 213 (2); 190 (23); 173 (7); 163 (29); 149 (100); 121 (18); 105 (31); 91 (33); 77 (9); 41 (5).

#### *N*-(2-Fluorophenyl)-2-(5-isopropyl-2-methylphenoxy)acetamide (**7**)

White crystalline solid (60 mg, 25% yield). mp: 82.1–83.8 °C. NMR, δ_H_ (400 MHz, CDCl_3_): 1.28 (d, 6H, *J* = 8.0 Hz, 2× CH_3_); 2.36 (s, 3H, CH_3_); 2.91 (sept, 1H, *J* = 8.0 Hz, CH); 4.68 (s, 2H, CH_2_); 6.74 (s, 1H, Ar-H_6_); 6.88 (d, 1H, *J* = 8.0 Hz, Ar-H_4_); 7.11–7.20 (m, 4H, Ar-H_3_, H_3′_, H_4′_, and H_5′_); 8.46 (t, 1H, *J* = 8.0 Hz, H_6′_); 8.85 (s, 1H, NH). δ_C_ (100 MHz, CDCl_3_): 15.8 (CH_3_); 24.1 (CH(CH_3_)_2_); 34.1 (CH(CH_3_)_2_); 67.4 (O–CH_2_); 109.9 (C_6_); 114.8 (d, *J* = 19.0 Hz, C_3′_); 119.9 (C_4_); 121.4 (C_1′_); 123.8 (C_2_); 124.7 (C_6′_); 131.0 (C_3_, C_4′_, and C_5′_); 148.6 (C_5_); 152.5 (d, *J* = 243.0 Hz, C_2′_); 155.0 (C_1_); 166.5 (C=O). MS, *m*/*z* (%): 301 (C_18_H_20_FNO_2_, [M]^+•^, 63); 190 (20); 163 (23); 149 (100); 135 (42); 124 (58); 111 (59); 91 (33); 77 (25); 65 (6); 41 (11).

#### *N*-(3-Fluorophenyl)-2-(5-isopropyl-2-methylphenoxy)acetamide (**8**)

White crystalline solid (144 mg, 60% yield), mp: 80.0–81.6 °C. NMR, δ_H_ (400 MHz, CDCl_3_): 1.27 (d, 6H, *J* = 8.0 Hz, 2× CH_3_); 2.36 (s, 3H, CH_3_); 2.91 (sept, 1H, *J* = 8.0 Hz, CH); 4.65 (s, 2H, CH_2_); 6.74 (s, 1H, Ar-H_6_); 6.89 (d, 1H, *J* = 8.0 Hz, Ar-H_4_); 7.16 (d, 1H, *J* = 8.0 Hz, H_3_); 7.24 (d, 1H, *J* = 8.0 Hz, H_5′_); 7.30–7.36 (m, 2H, H_2′_ and H_4′_); 7.60 (d, 1H, *J* = 8.0 Hz, H_6′_); 8.46 (s, 1H, NH). δ_C_ (100 MHz, CDCl_3_): 16.0 (CH_3_); 24.0 (CH(CH_3_)_2_); 34.0 (CH(CH_3_)_2_); 67.8 (O–CH_2_); 107.3 (d, *J* = 26.0 Hz, C_4′_); 110.5 (C_6_); 111.6 (d, *J* = 21.0 Hz, C_2′_); 115.2 (d, *J* = 3.0 Hz, C_6′_); 120.2 (C_4_); 123.7 (C_2_); 130.3 (d, *J* = 9.0 Hz, C_5′_); 131.1 (C_3_); 138.4 (d, *J* = 11.0 Hz, C_1′_); 148.8 (C_5_); 155.1 (C_1_); 164.3 (d, *J* = 244.0 Hz, C_3′_); 166.7 (C=O). MS, *m*/*z* (%): 301 (C_18_H_20_FNO_2_, [M]^+•^, 61); 190 (15); 163 (22); 149 (100); 124 (34); 111 (26); 91 (28); 77 (14); 57 (4); 41 (9).

#### *N*-(4-Fluorophenyl)-2-(5-isopropyl-2-methylphenoxy)acetamide (**9**)

White crystalline solid (115 mg, 40% yield), mp: 108.4–110.1 °C. NMR, δ_H_ (400 MHz, CDCl_3_): 1.27 (d, 6H, *J* = 8.0 Hz, 2× CH_3_); 2.35 (s, 3H, CH_3_); 2.91 (sept, 1H, *J* = 8.0 Hz, CH); 4.65 (s, 2H, CH_2_); 6.74 (s, 1H, Ar-H_6_); 6.89 (d, 1H, *J* = 8.0 Hz, Ar-H_4_); 7.08 (m, 2H, H_3′_ and H_5′_); 7.16 (d, 1H, *J* = 8.0 Hz, Ar-H_3_); 7.59 (m, 2H, H_2′_ and H_6′_); 8.38 (s, 1H, NH). δ_C_ (100 MHz, CDCl_3_): 16.0 (CH_3_); 24.1 (CH(CH_3_)_2_); 34.0 (CH(CH_3_)_2_); 67.8 (O–CH_2_); 110.4 (C_6_); 115.8 (d, *J* = 22.5 Hz, C_3′_ and C_5′_); 120.1 (C_4_); 121.8 (d, *J* = 7.8 Hz, C_2′_ and C_6′_); 123.8 (C_2_); 131.1 (C_3_); 132.9 (d, *J* = 3.2 Hz, C_1′_); 148.7 (C_5_); 155.2 (C_1_); 159.7 (d, *J* = 243.0 Hz, C_4'_); 166.6 (C=O). MS, *m*/*z* (%): 301 (C_18_H_20_FNO_2_, [M]^+•^, 65); 190 (19); 163 (27); 149 (100); 135 (32); 124 (41); 111 (41); 91 (22); 83 (10); 65 (4); 41 (6).

#### 2-(5-Isopropyl-2-methylphenoxy)-*N*-phenylacetamide (**10**)

White crystalline solid (91 mg, 40% yield), mp: 86.2–87.5 °C. NMR, δ_H_ (400 MHz, CDCl_3_): 1.27 (d, 6H, *J* = 7.2 Hz, 2× CH_3_); 2.36 (s, 3H, CH_3_); 2.91 (sept, 1H, *J* = 7.2 Hz, CH); 4.66 (s, 2H, CH_2_); 6.75 (s, 1H, Ar-H_4_); 6.86 (d, 1H, *J* = 7.6 Hz, Ar-H_6_); 7.15–7.21 (m, 2H, *J* = 8.0 Hz, H_3′_ and H_4′_); 7.40 (t, 2H, *J* = 7.6 Hz, H_3′_ and H_5′_); 7.61–7.63 (d, 2H, *J* = 7.6 Hz, H_2′_ and H_6′_); 8.41 (s, 1H, NH). δ_C_ (100 MHz, CDCl_3_): 16.0 (CH_3_); 24.1 (CH(CH_3_)_2_); 34.1 (CH(CH_3_)_2_); 67.8 (O–CH_2_); 110.4 (C_6_); 120.0 (C_2′_ and C_6′_); 120.8 (C_4_); 124.9 (C_2_); 129.2 (C_3′_ and C_5′_); 131.0 (C_3_); 136.9 (C_1′_); 148.7 (C_5_); 155.2 (C_1_); 166.6 (C=O). MS, *m*/*z* (%): 284 ([M + 1]^+•^, 17); 283 (C_19_H_21_NO_2_, [M]^+•^, 81); 163 (28); 149 (100); 135 (51); 121 (29); 106 (27); 93 (41); 77 (27); 65 (15); 51 (4).

### X-ray Crystallography C_18_H_20_FNO_2_ (**9**)

Suitable crystals for X-ray diffraction of C_18_H_20_FNO_2_ (**9**) were mounted in a glass fiber. Data were collected at room temperature on a XtaLAB Synergy Dualflex diffractometer equipped with HyPix detector, using Mo Kα radiation from a microfocus X-ray tube. Data were collected at a resolution of 0.8 Å. Final unit cell parameters were based on the fitting of all reflection positions and collected data were merged considering space group *P*3_1_ (*R*_int_ = 0.0300). The crystal structure was solved by direct methods and refined on *F*^2^ by full-matrix least-squares using the SHELX-86^38,39^ program in a WinGX Plataforma.^40^ The structure of the C_18_H_20_FNO_2_ unit was initially found and refined considering anisotropic displacement for all but hydrogen atoms. Hydrogen atoms were fixed geometrically. Molecular graphics and packing figures were obtained from Ortep^41^ and Mercury,^42^ respectively.

The crystals data, data collection procedures, structure determination methods and refinement results are summarized in Table S1 (Supporting Information).

### Cytotoxicity Assay in Macrophages

RAW 264.7 cells were cultured in Roswell Park Memorial Institute (RPMI) medium, pH 7.2, supplemented with 10% (v/v) heat-inactivated fetal bovine serum (FBS) (Gibco), 100 μg mL^–1^ penicillin (Sigma), 2 g L^–1^ sodium bicarbonate, 2 mM l-glutamine, and 25 mM HEPES, and maintained at 37 °C in a humidified incubator with 5% CO_2_ atmosphere.^27^ The cells were then seeded into sterile 96-well plates at a density of 5 × 10^4^ cells/well, followed by the addition of the compounds at a concentration of 10 μM for screening, and in a range of 200 μM to 1.56 μM for the determination of the 50% cytotoxic concentration (CC_50_). Controls included 1% DMSO and amphotericin B at 3.125 μg mL^–1^, with 48 h of treatment. Cell viability was assessed using 1 mM resazurin, with subsequent readings performed on a Spectramax spectrophotometer at wavelengths of 570 and 600 nm.

### Cytotoxicity Assay in Vero Cells

The Vero cell (ATCC, CCL-81) was cultured in Dulbecco’s modified Eagle medium (DMEM), pH 7.3, supplemented with 10% FBS (v/v) and a penicillin/streptomycin solution (0.1 mg mL^–1^/100 U mL^–1^). The cells were incubated at 37 °C in a humidified atmosphere with 5% CO_2_. The MTT reduction assay (3-(4,5-dimethylthiazol-2-yl)-2,5-diphenyltetrazolium bromide) was adapted from Mosmann^43^ to evaluate cytotoxicity. For this assay, 1 × 10^4^ cells per well were seeded in 96-well plates and incubated overnight until reaching approximately 80% confluence. Wells were treated with different compound concentrations (500 to 7.8 μM) in triplicate. The culture medium was replaced with 0.5 mg mL^–1^ MTT solution, and the plate was incubated for 3 h. The resulting formazan crystals were solubilized in DMSO, and absorbance was measured using a Spectramax spectrophotometer at a wavelength of 540 nm to assess cell viability.

### Leishmanicidal Activity Assays

*L. braziliensis* MHOM/BR/75/M2904 was cultured in Grace’s insect medium (pH 6.5) supplemented with 10% (v/v) heat-inactivated fetal bovine serum (FBS) (Gibco), 100 μg mL^–1^ penicillin (Sigma), and 2 mM l-glutamine, maintained at 25 °C in a B.O.D. incubator. Using the stationary phase culture on the seventh day, 2.0 × 10^6^*Leishmania*/well were transferred to a 96-well plate containing 1.0 × 10^5^ preadhered macrophages/well, following an adapted infection protocol.^44^ After applying the compounds at a concentration of 10 μM, 1% DMSO and amphotericin B (3.125 μg mL^–1^) were used as controls. Treatments were conducted for 48 h, followed by macrophage lysis using 0.1% SDS solution, which was subsequently neutralized with complete Grace’s medium. To recover promastigote forms, the plate was incubated for 5 days. Fluorescence was measured using a Spectramax fluorometer, with excitation at 490 nm and emission at 520 nm.

### Statistical Analyzes

All statistical analyses were performed using GraphPad Prism 8 software (GraphPad Software, San Diego, CA, USA). Differences between groups were evaluated using one-way analysis of variance (ANOVA), followed by Dunnett’s posthoc test for multiple comparisons. A *p*-value of less than 0.05 was considered statistically significant. Data are presented as mean ± standard deviation (SD).

## Supporting Information Available

FTIR and mass spectra of carvacroxyacetic acid, as well as ^1^H and ^13^C NMR spectra and mass spectra of all synthesized *N*-phenyl-2-phenoxyacetamides (PDF)
