# Supplementary material for: Synthesis and Antileishmanial Evaluation of N-Phenyl-2-phenoxyacetamides Derived from Carvacrol
Source: ACS Omega. 2025 Apr 23;10(17):17565–75. doi: 10.1021/acsomega.4c11359 (PMC12059944; doi:10.1021/acsomega.4c11359)
Supplement: Supplementary file 1 — ao4c11359_si_001.pdf [file ao4c11359_si_001.pdf]

Synthesis and Antileishmanial Evaluation of *N*-Phenyl-2-phenoxyacetamides Derived from Carvacrol

Rayane Luiza de Carvalho[1**], Alícia da Conceição Terbutino da Silva[2**], Bianca Muniz Lacerda Ventura[1**], Sara Andrade Machado Godoy[1], Olívia Géraldine Audrey Avome Nguema[1], Juliana Lopes Rangel Fietto[1], Christiane Mariotini-Moura[3], Michelle Dias de Oliveira Teixeira[1], Raphael de Souza Vasconcellos[1], Angel Amado Recio Despaigne[2], Bruna Vidal Paes[4], Bernardo Lages Rodrigues[4], Ulisses Alves Pereira[5], and Patrícia Fontes Pinheiro[2*]

[1]Department of Biochemistry and Molecular Biology, Federal University of Viçosa, Avenida Peter Henry Rolfs, s/n, 36570-900, Viçosa, MG, Brazil.

[2]Department of Chemistry, Federal University of Viçosa, Avenida Peter Henry Rolfs, s/n, 36570-900, Viçosa, MG, Brazil.

[3]Department of Medicine and Nursing, Federal University of Viçosa, Avenida Peter Henry Rolfs, s/n, 36570-900, Viçosa, MG, Brazil.

[4]Department of Chemistry, Federal University of Minas Gerais, 1270-901, Belo Horizonte, MG, Brazil.

[5]Federal University of Minas Gerais, Montes Claros Regional Campus, Institute of Agricultural Sciences, Avenida Universitária 1000, Bairro Universitário, 39404-547, Montes Claros, MG, Brazil.

The authors (**) contributed equally. *Author to whom correspondence should be addressed. Telephone: (55) 031 36126640 and (55) 028 35528621. E-mail: patricia.pinheiro@ufv.br.

Supporting Information

FTIR spectrum of carvacroxyacetic acid **Figure S1**

Mass spectra of carvacroxyacetic acid **Figure S2**

^{1}H and ^{13}C NMR spectrum of all novel synthesized compounds **Figures S3-S22**

Mass spectra of all novel synthesized compounds **Figures S23-S32**

Table S1. Crystal data, data collection parameters, and refinement results for $C_{18}H_{20}FNO_2$

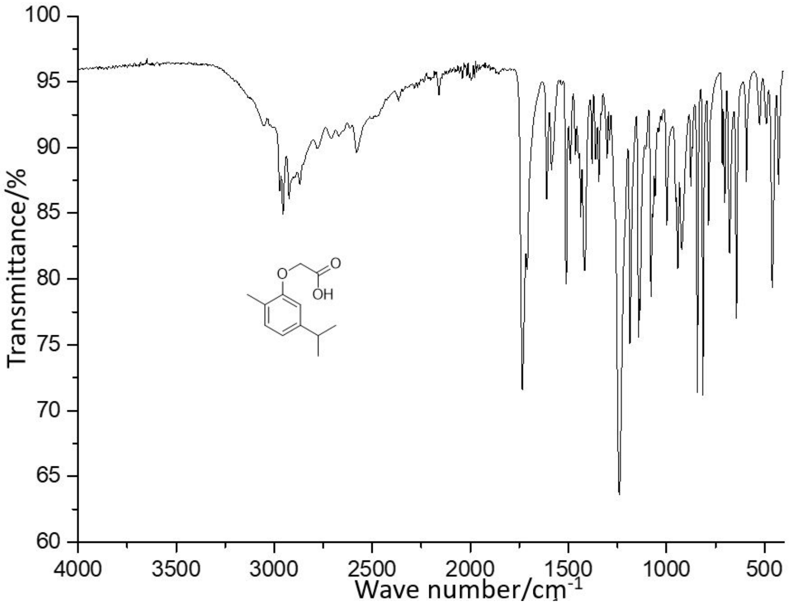

Figure S1. FTIR spectrum of carvacroxyacetic acid.

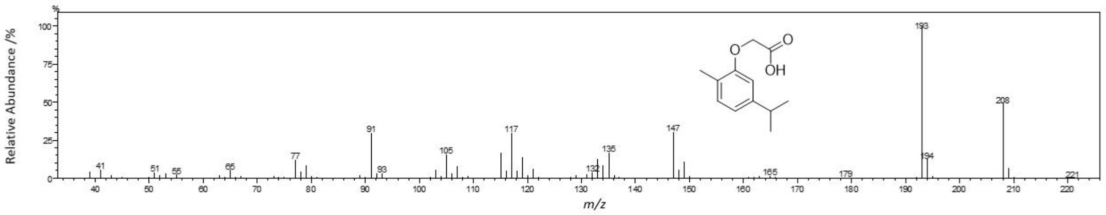

Figure S2. Mass spectra of carvacroxyacetic acid.

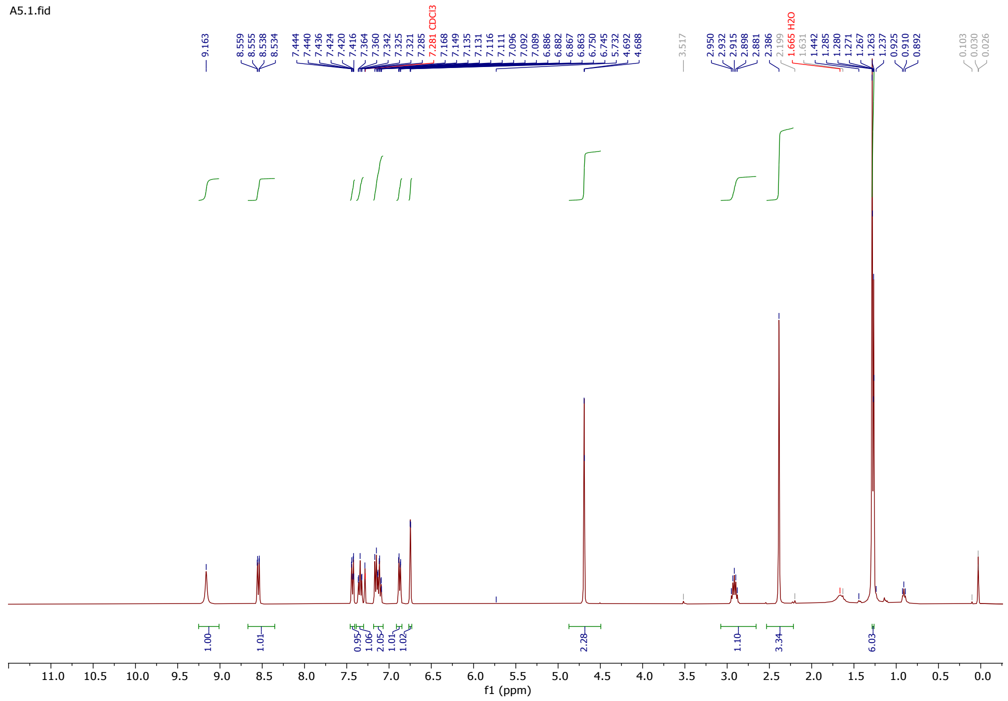

Figure S3. ^{1}H NMR spectrum of *N*-(2-chlorophenyl)-2-(5-isopropyl-2-methylphenoxy)acetamide (**1**).

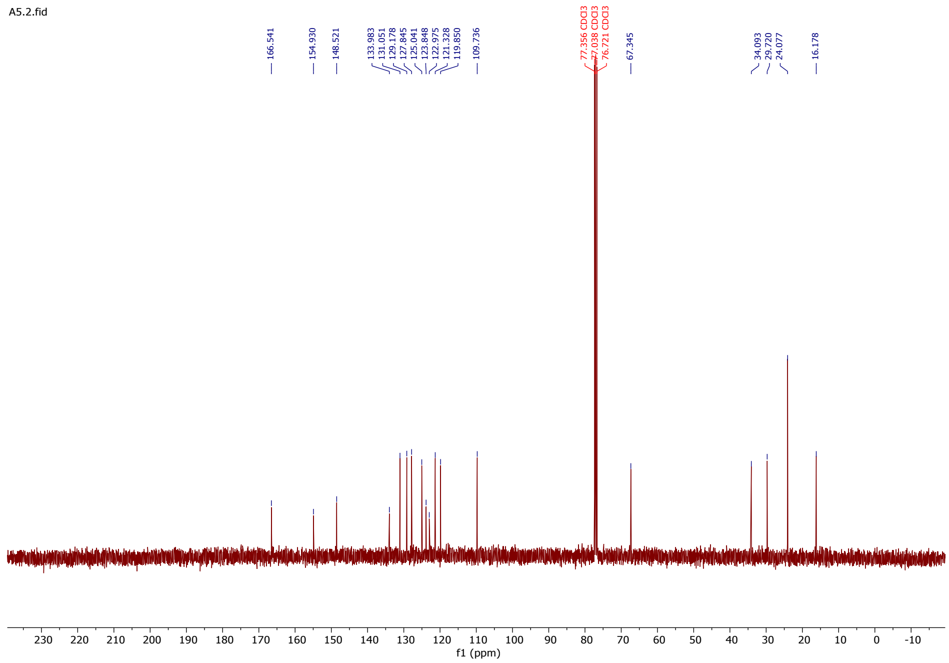

Figure S4. ^{13}C NMR spectrum of *N*-(2-chlorophenyl)-2-(5-isopropyl-2-methylphenoxy)acetamide (**1**).

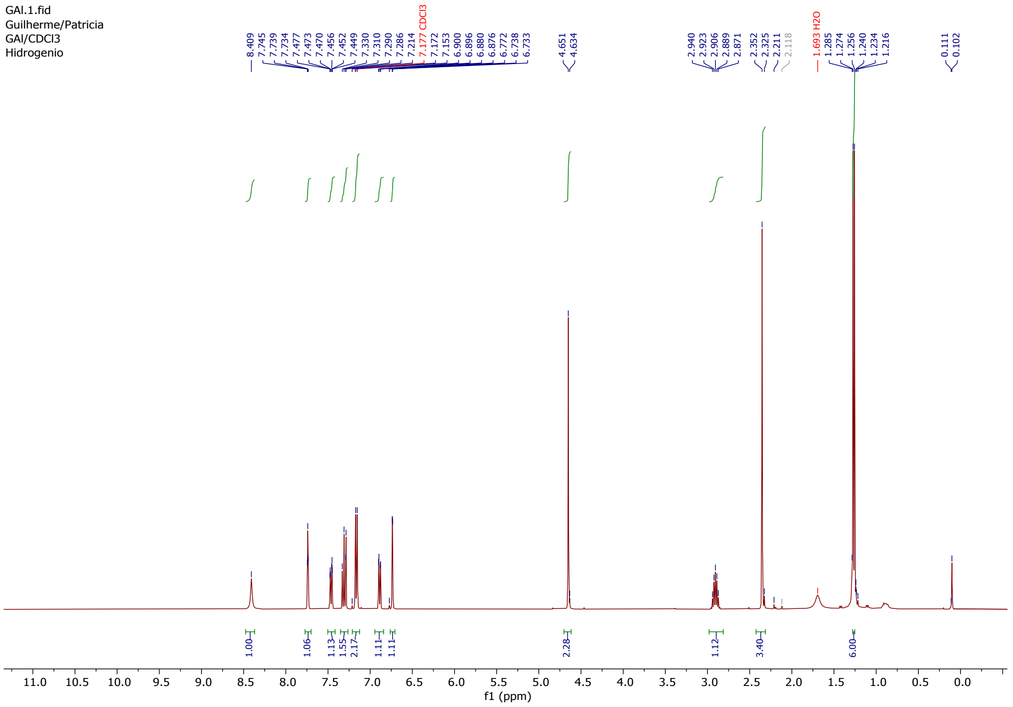

Figure S5. ^{1}H NMR spectrum of *N*-(3-chlorophenyl)-2-(5-isopropyl-2-methylphenoxy)acetamide (**2**).

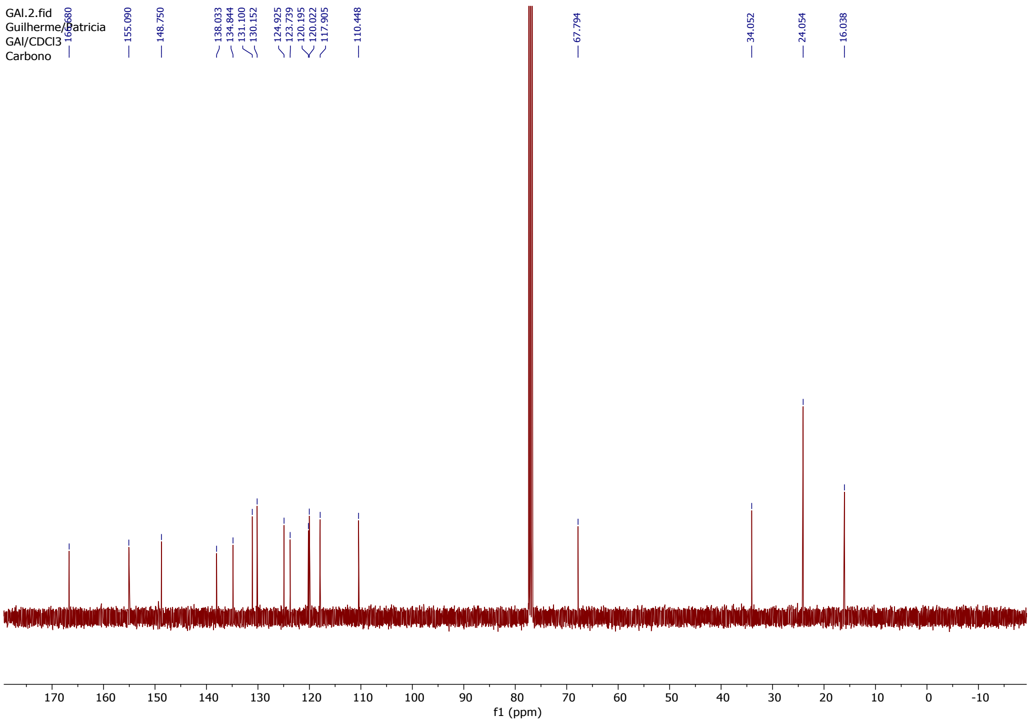

Figure S6. ^{13}C NMR spectrum of *N*-(3-chlorophenyl)-2-(5-isopropyl-2-methylphenoxy)acetamide (**2**).

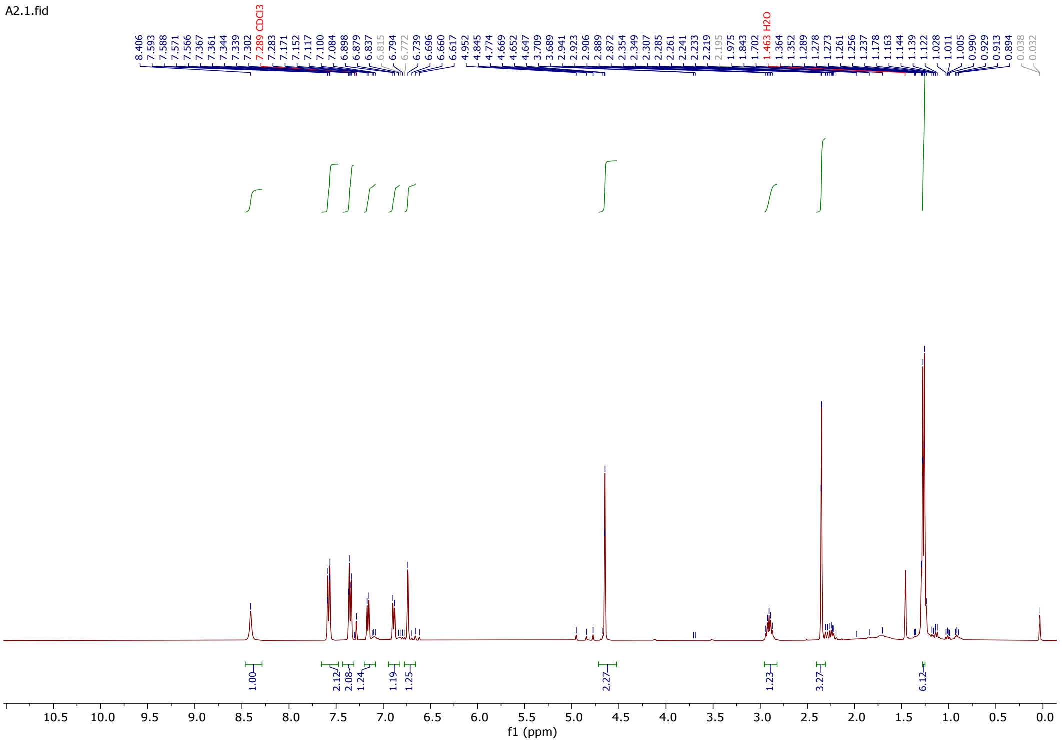

Figure S7. ^{1}H NMR spectrum of *N*-(4-chlorophenyl)-2-(5-isopropyl-2-methylphenoxy)acetamide (**3**).

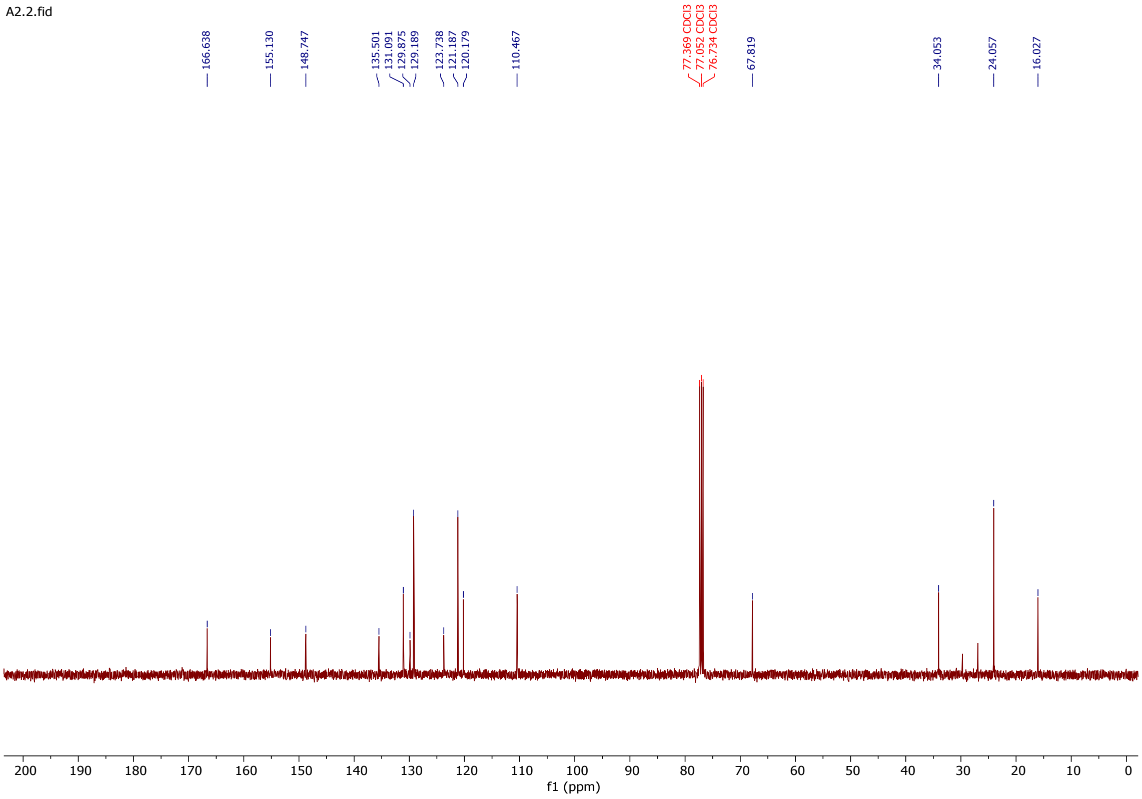

Figure S8. ^{13}C NMR spectrum of *N*-(4-chlorophenyl)-2-(5-isopropyl-2-methylphenoxy)acetamide (**3**).

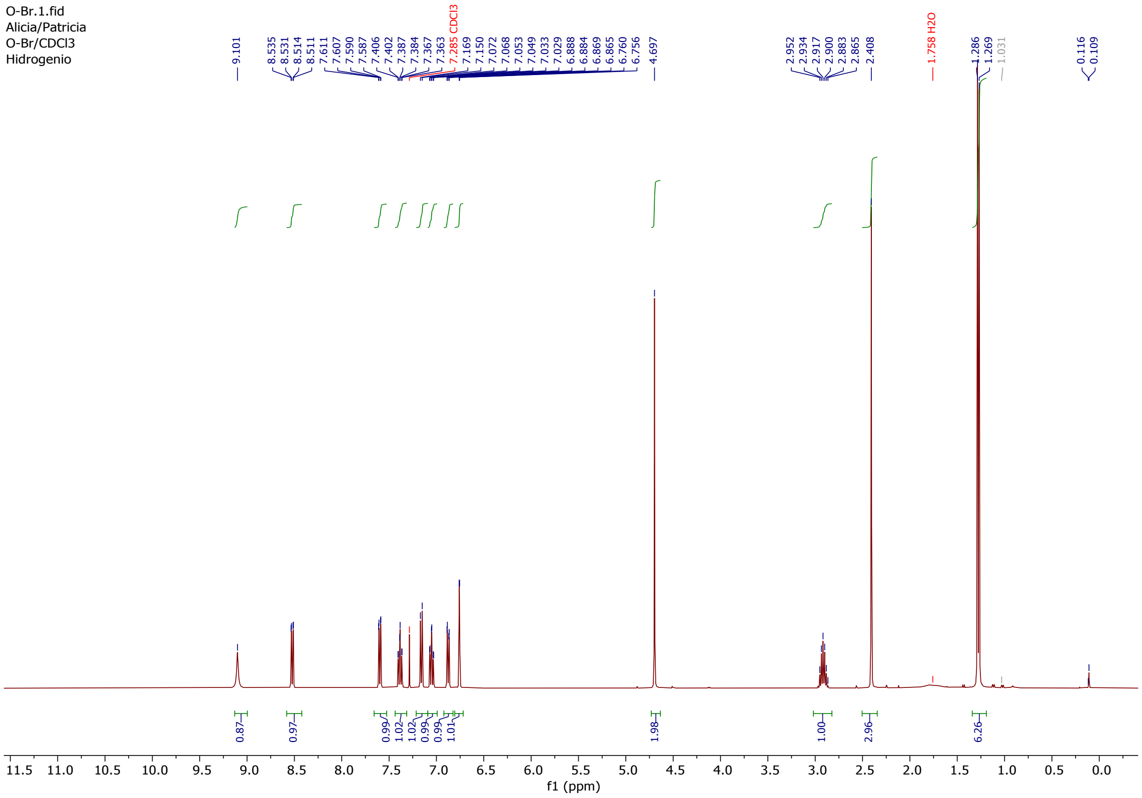

Figure S9. ^{1}H NMR spectrum of *N*-(2-bromophenyl)-2-(5-isopropyl-2-methylphenoxy)acetamide (**4**).

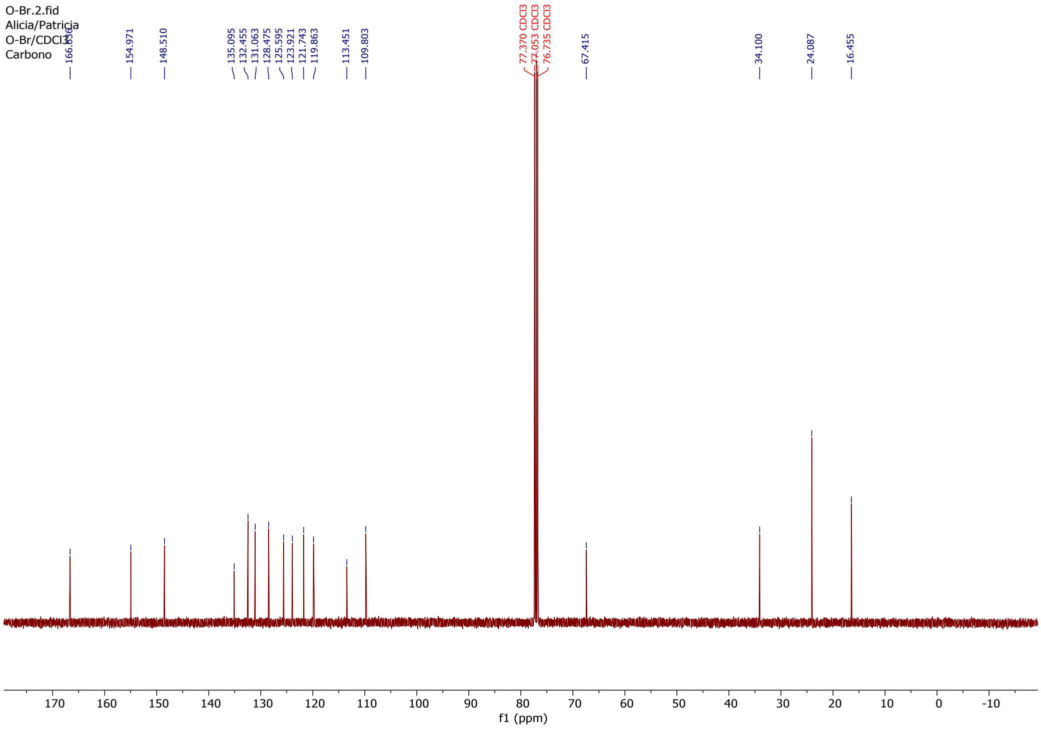

Figure S10. ^{13}C NMR spectrum of 2 *N*-(2-bromophenyl)-2-(5-isopropyl-2-methylphenoxy)acetamide (**4**).

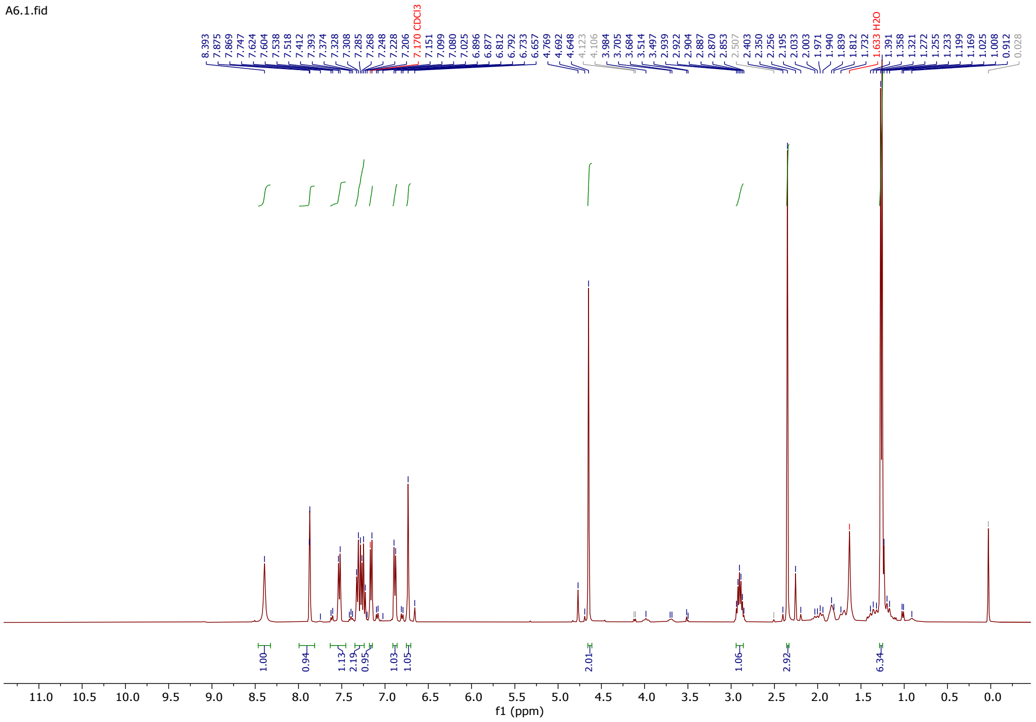

Figure S11. ^{1}H NMR spectrum of *N*-(3-bromophenyl)-2-(5-isopropyl-2-methylphenoxy)acetamide (**5**).

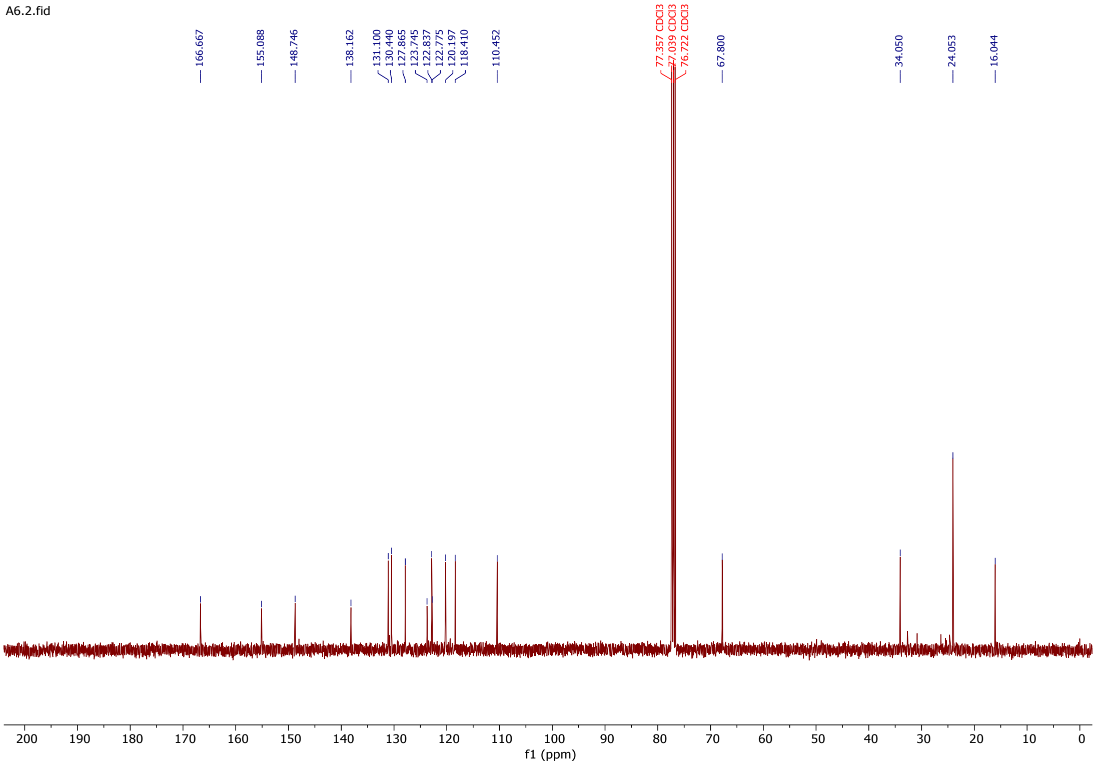

Figure S12. ^{13}C NMR spectrum of *N*-(3-bromophenyl)-2-(5-isopropyl-2-methylphenoxy)acetamide (**5**).

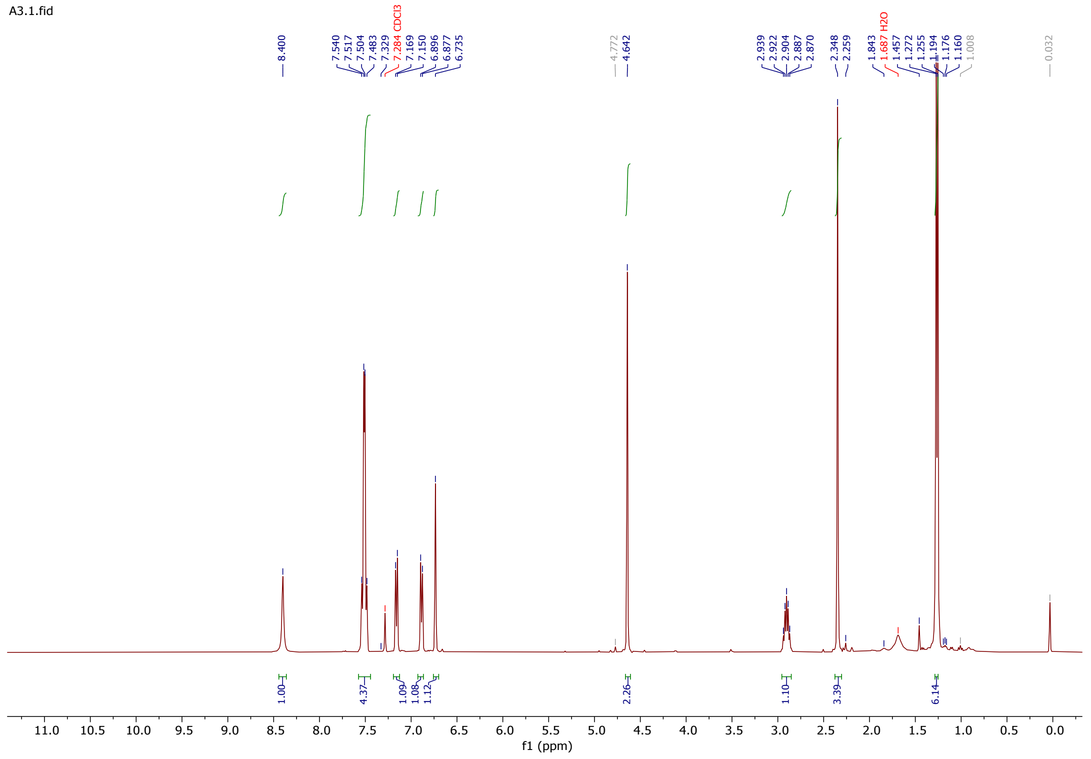

Figure S13. ^{1}H NMR spectrum of *N*-(4-bromophenyl)-2-(5-isopropyl-2-methylphenoxy)acetamide (**6**).

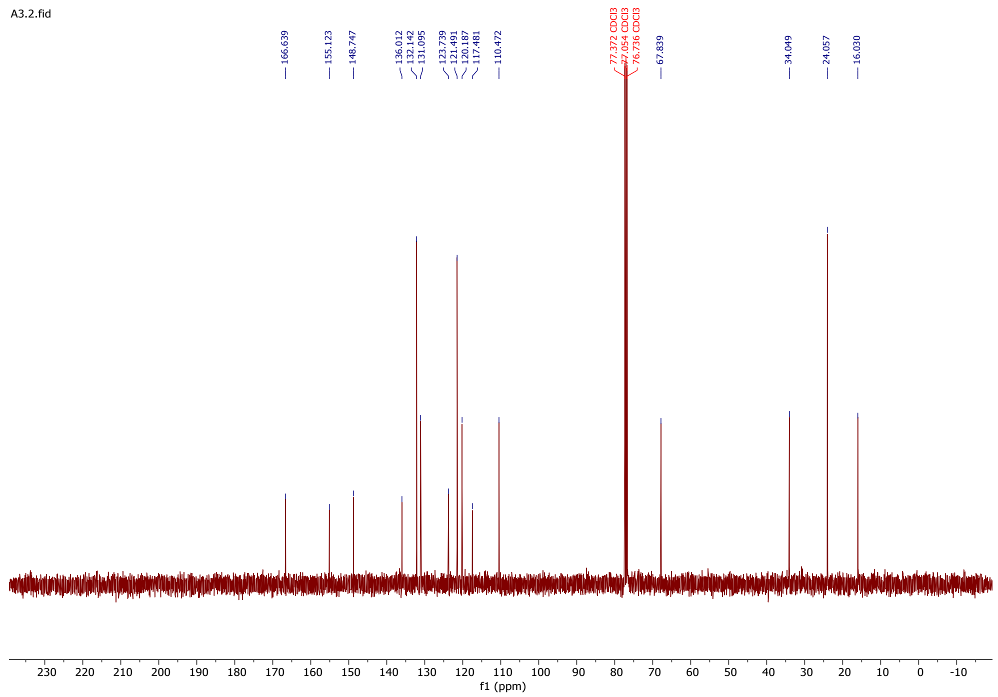

Figure S14. ^{13}C NMR spectrum of *N*-(4-bromophenyl)-2-(5-isopropyl-2-methylphenoxy)acetamide (**6**).

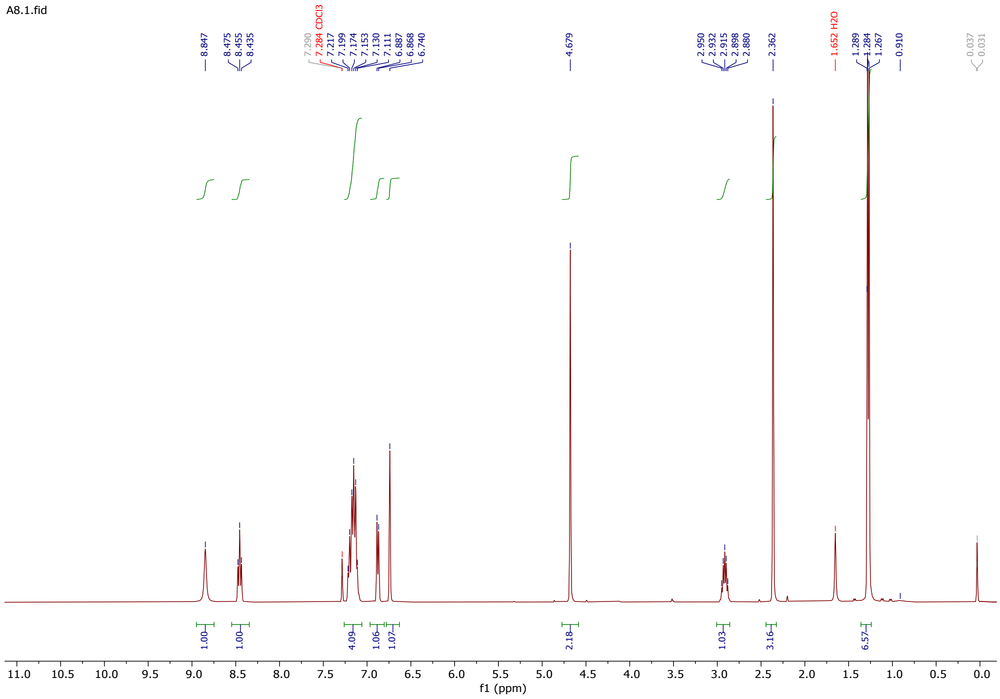

Figure S15. ^{1}H NMR spectrum of *N*-(2-fluorophenyl)-2-(5-isopropyl-2-methylphenoxy)acetamide (**7**).

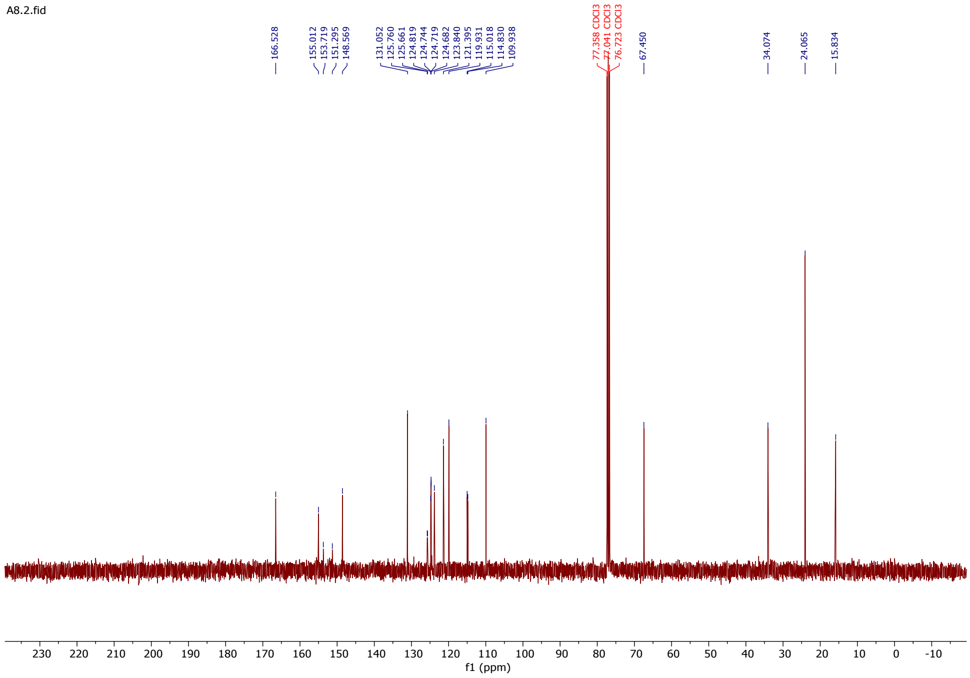

Figure S16. ^{13}C NMR spectrum of *N*-(2-fluorophenyl)-2-(5-isopropyl-2-methylphenoxy)acetamide (**7**).

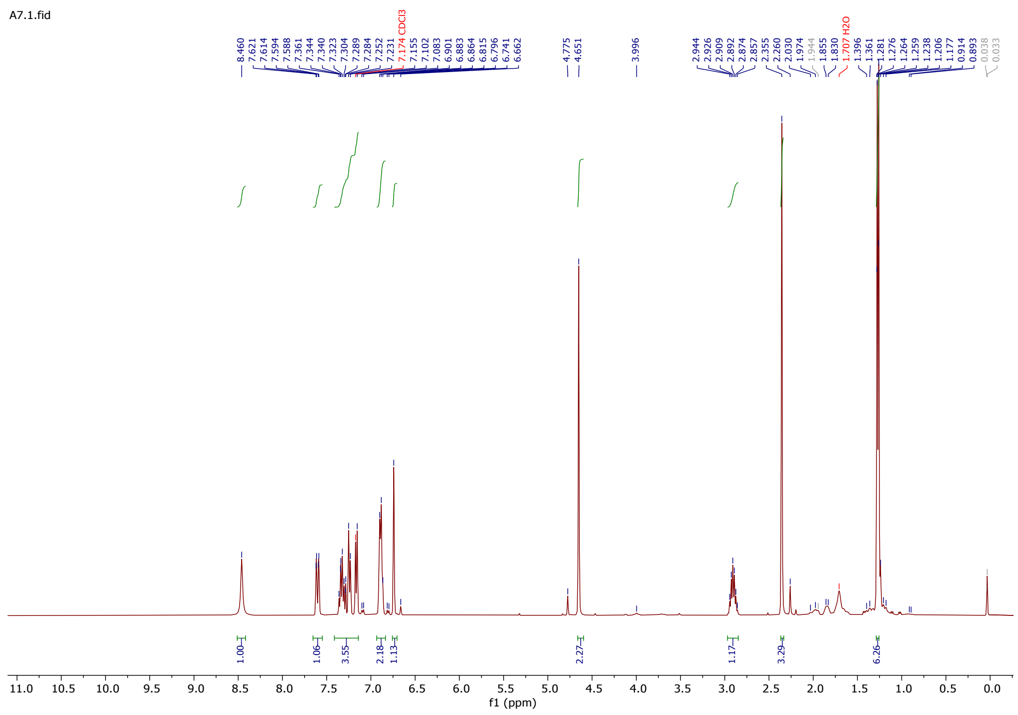

Figure S17. ^{1}H NMR spectrum of *N*-(3-fluorophenyl)-2-(5-isopropyl-2-methylphenoxy)acetamide (**8**).

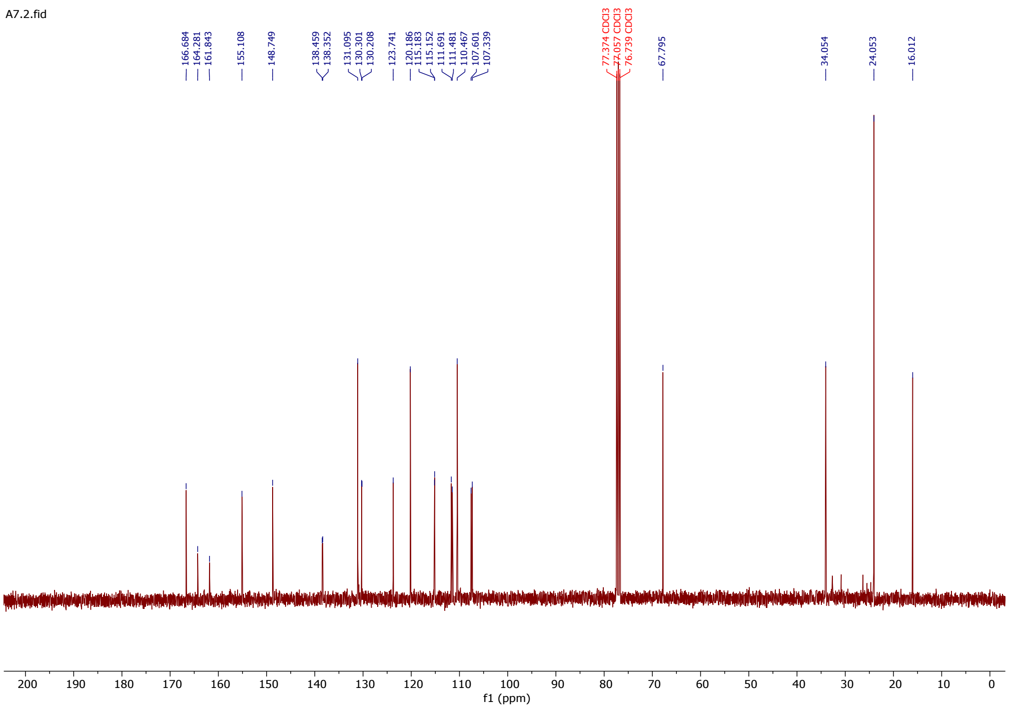

Figure S18. ^{13}C NMR spectrum of *N*-(3-fluorophenyl)-2-(5-isopropyl-2-methylphenoxy)acetamide (**8**).

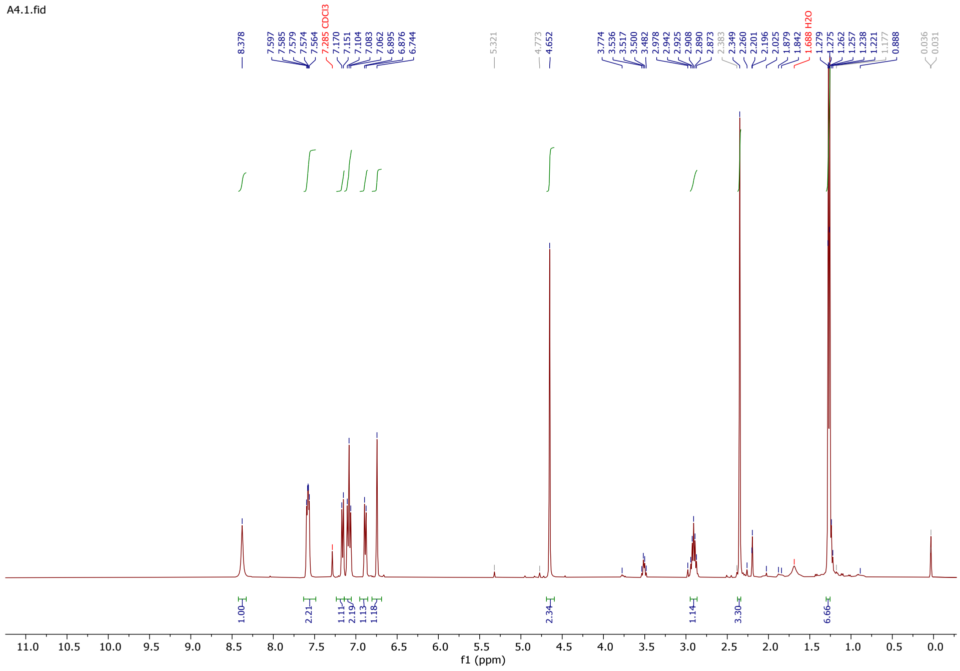

Figure S19. ^{1}H NMR spectrum of *N*-(4-fluorophenyl)-2-(5-isopropyl-2-methylphenoxy)acetamide (**9**).

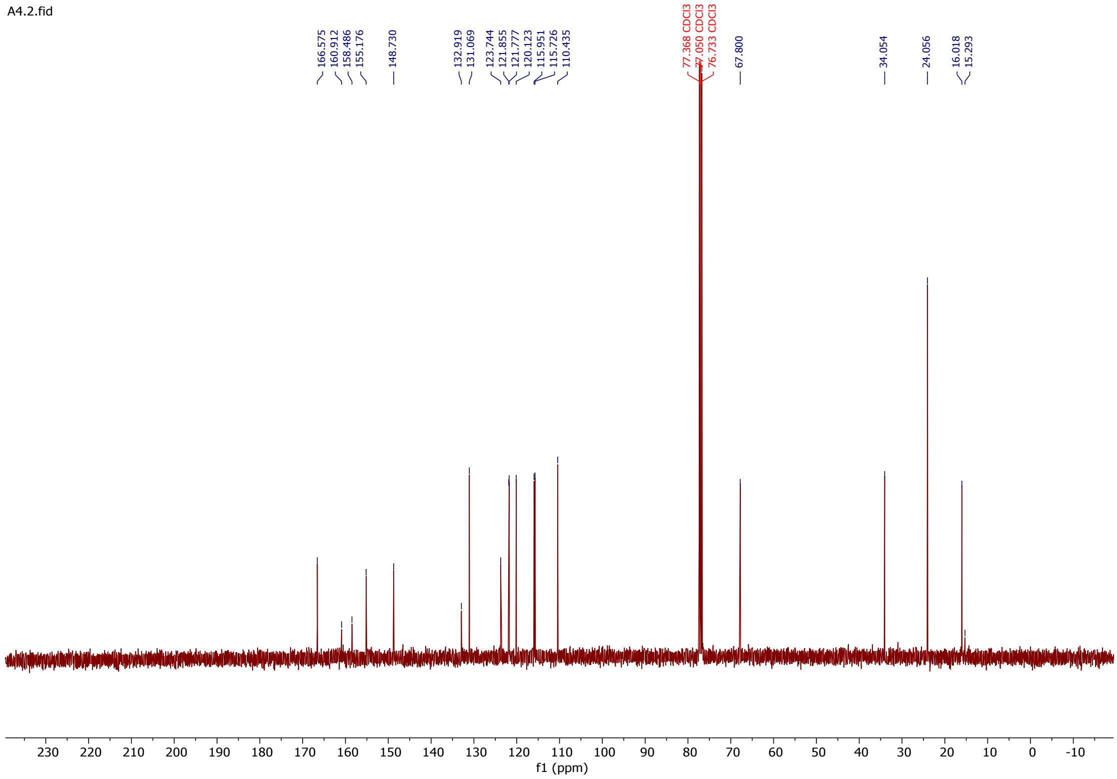

Figure S20. ^{13}C NMR spectrum of *N*-(4-fluorophenyl)-2-(5-isopropyl-2-methylphenoxy)acetamide (**9**).

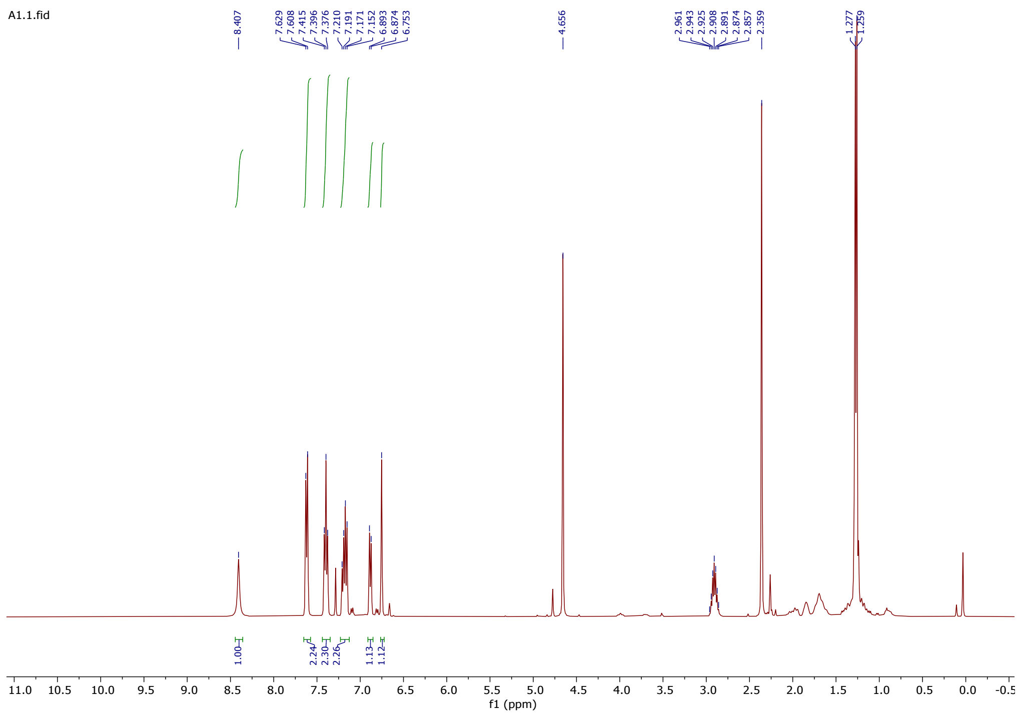

Figure S21. ^{1}H NMR spectrum of 2-(5-isopropyl-2-methylphenoxy)-N-phenylacetamide (**10**).

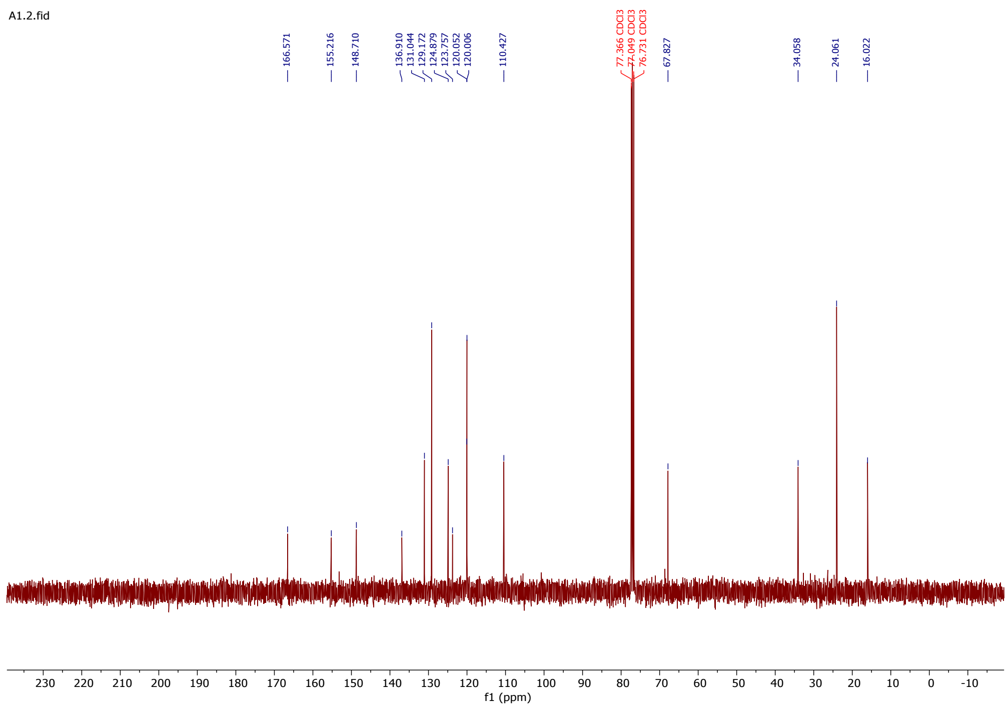

Figure S22. ^{13}C NMR spectrum of 2-(5-isopropyl-2-methylphenoxy)-N-phenylacetamide (**10**).

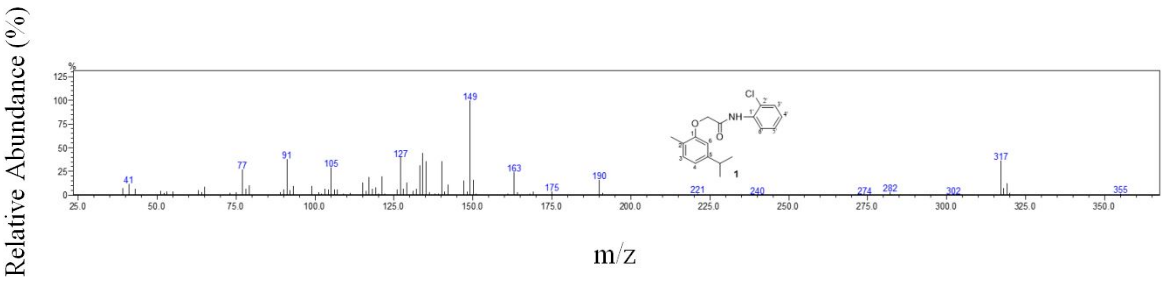

Figure S23. Mass spectrum of *N*-(2-chlorophenyl)-2-(5-isopropyl-2-methylphenoxy)acetamide (**1**).

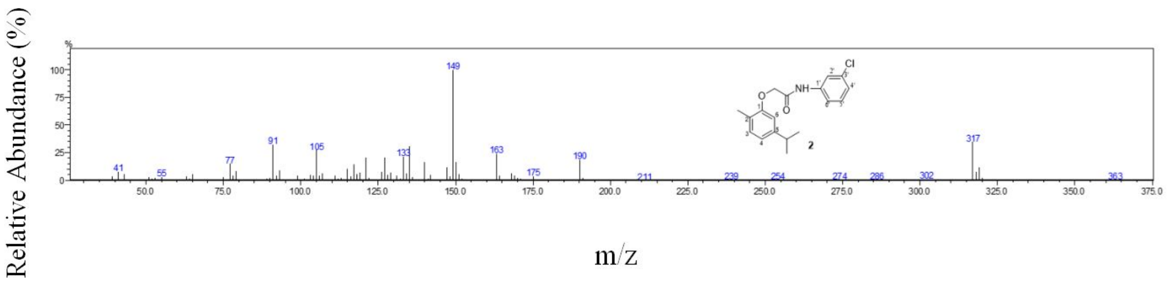

Figure S24. Mass spectrum of *N*-(3-chlorophenyl)-2-(5-isopropyl-2-methylphenoxy)acetamide (**2**).

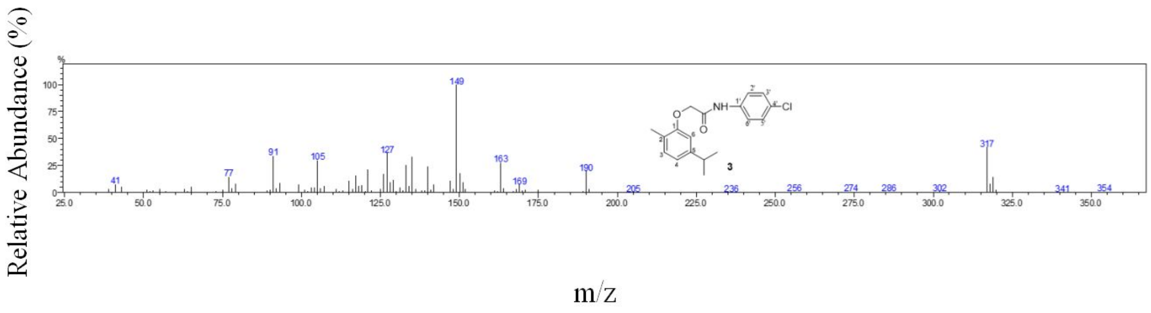

Figure S25. Mass spectrum of *N*-(4-chlorophenyl)-2-(5-isopropyl-2-methylphenoxy)acetamide (**3**).

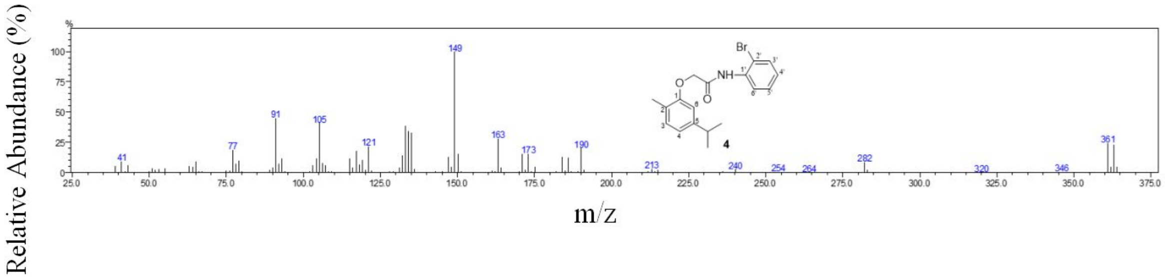

Figure S26. Mass spectrum of *N*-(2-bromophenyl)-2-(5-isopropyl-2-methylphenoxy)acetamide (**4**).

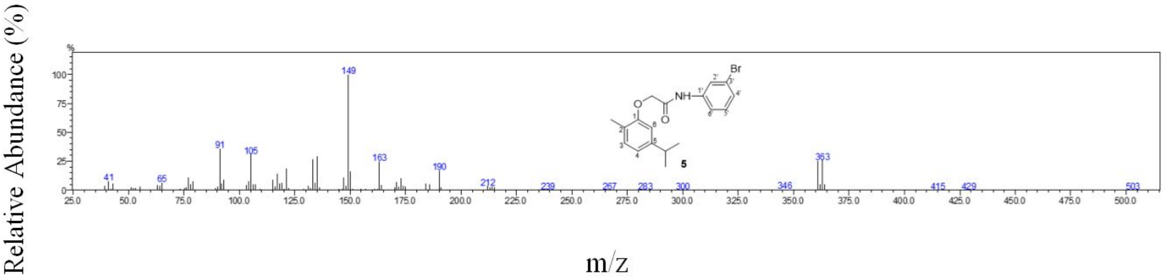

Figure S27. Mass spectrum of *N*-(3-bromophenyl)-2-(5-isopropyl-2-methylphenoxy)acetamide (**5**).

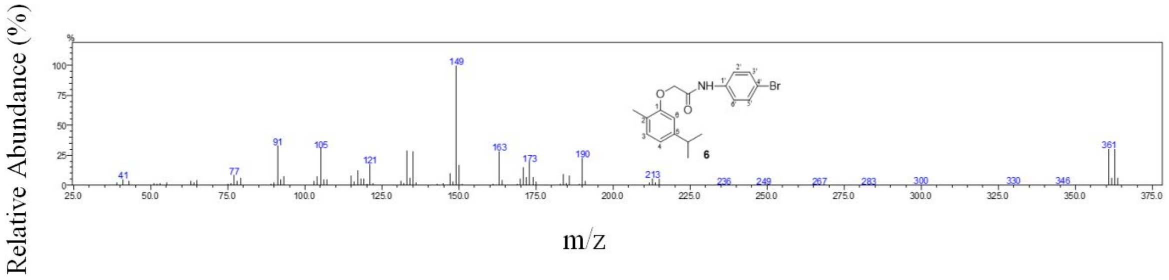

Figure S28. Mass spectrum of *N*-(4-bromophenyl)-2-(5-isopropyl-2-methylphenoxy)acetamide (**6**).

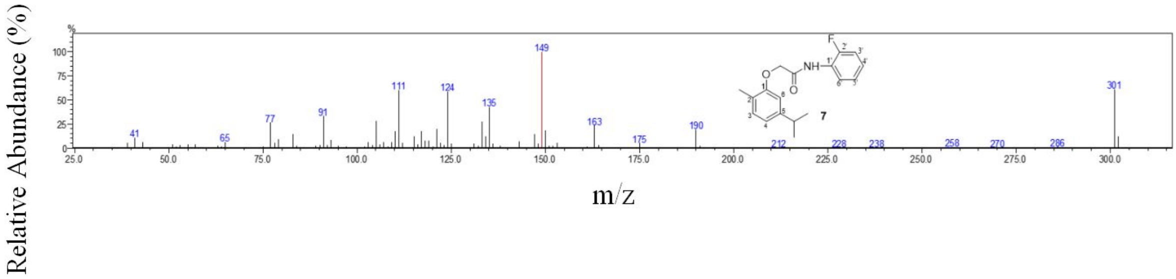

Figure S29. Mass spectrum of *N*-(2-fluorophenyl)-2-(5-isopropyl-2-methylphenoxy)acetamide (**7**).

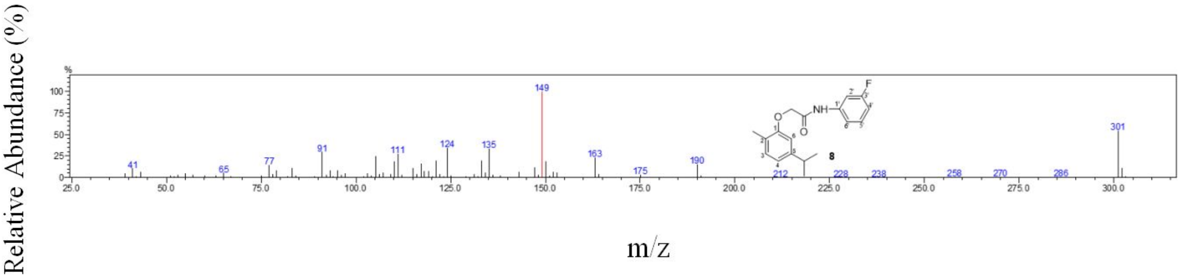

Figure S30. Mass spectrum of *N*-(3-fluorophenyl)-2-(5-isopropyl-2-methylphenoxy)acetamide (**8**).

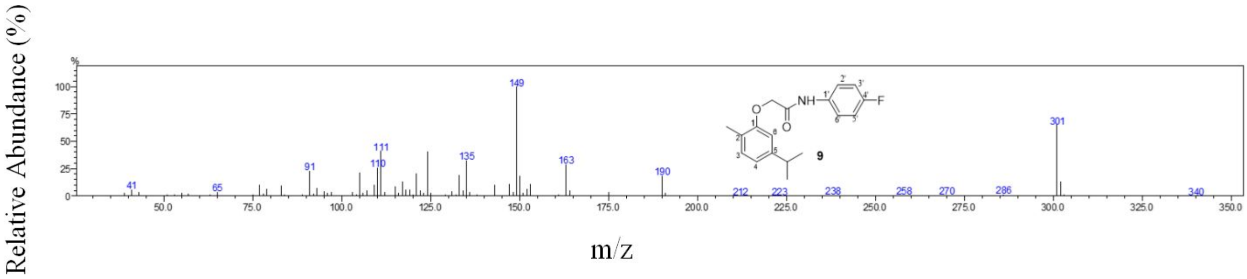

Figure S31. Mass spectrum of *N*-(4-fluorophenyl)-2-(5-isopropyl-2-methylphenoxy)acetamide (**9**).

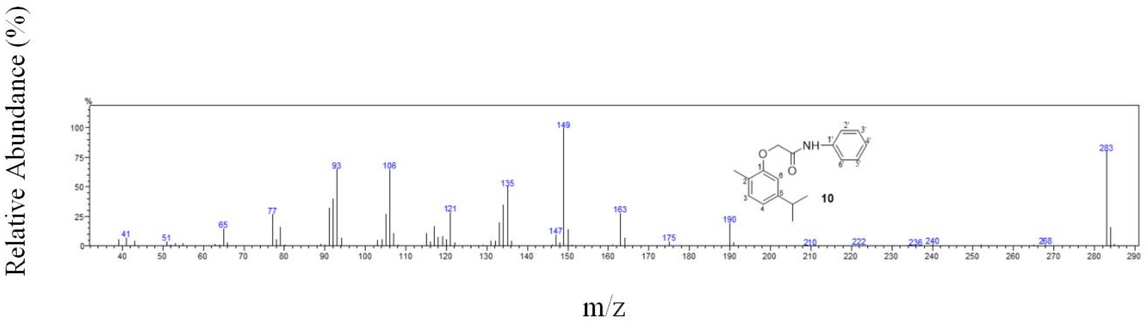

Figure S32. Mass spectrum of 2-(5-isopropyl-2-methylphenoxy)-*N*-phenylacetamide (**10**).

Table S1. Crystal data, data collection parameters, and refinement results for $C_{18}H_{20}FNO_2$

Empirical formula		$C_{18}H_{20}FNO_2$
Formula weight		567.44
Crystal system		Trigonal
Space group		$P\ 3_1$
Diffractometer		Rigaku Synergy
Wavelength, Å		0.71073 Å
Unit cell dimensions	*a*, Å	10.1625(2)
	b, Å	10.1625(2)
	c, Å	13.7703(3)
	α, θ	90
	β, θ	90
	γ, θ	120
Volume, $Å^3$		1231.61(6)
Z, Density calc., Mg/m^3		2, 1.530
Absorption coefficient, mm^{-1}		0.126
F(000)		578
Crystal size, mm		0.85 x 0.20 x 0.16 mm^3
θ range for data coll., º		2.314-30.644
Index range, θ		$-14 \le h \le 14$
		$-14 \le k \le 12$
		$-19 \le l \le 18$
Completeness		θ = 25.242º
		100.0%
Goodness-of-fit on F^2		1.079
Reflec. collect./unique (R_{int})		19710/4257 (0.0300)
Data/restraints/parameters		4257/1/199
Observed reflections, $I > 2\sigma(I)$		3766
Final R indices [$I > 2\sigma(I)$]		R1 = 0.0357, wR2 = 0.0883
R indices (all data)		R1 = 0.0411, wR2 = 0.0908
Large peak & hole, e $Å^{-3}$		0.119/-0.162
